# Gas-entry pressure impact on the evaluation of hydrogen migration at different scales of a deep geological disposal of radioactive waste

**DOI:** 10.1038/s41598-024-56454-y

**Published:** 2024-03-14

**Authors:** Zakaria Saâdi

**Affiliations:** Institut de Radioprotection et de Sûreté Nucléaire (IRSN), PSE-ENV, SPDR, UEMIS, 92262 Fontenay-aux-Roses, France

**Keywords:** Hydrology, Computational science

## Abstract

Although the importance of gas-entry pressure in simulating two-phase liquid–gas flow in porous media has been studied at the column and borehole scales, its impact on the simulation of transient hydraulic-gas at different scales of a deep geological repository of radioactive waste (DGR) in low permeability clay rock during the post-closure phase has not yet been studied. The purpose of this work is to show that neglecting this phenomenon can lead to underestimation of the maximum gas pressure and water–gas fluxes simulated within the host rock and backfilled drift network. This could impact the performance of the engineered barrier system of a DGR. Simulations performed for a high-level waste disposal cell and for a simplified repository composed of hundreds of disposal cells situated in a clay host rock, show that gas preferentially migrates through the DGR components with low capillary entry pressures, such as the excavation damaged zone (Refers to the zone where fractures develop due to failure of the rock mass around galleries after tunneling) (EDZ), the engineered barriers materials (backfill, bentonite-plug…) and interfaces between the EDZ and these materials. Such a result could have significant consequences on the performance of a repository, due to the accumulation of gas in the drift network and high increase of gas pressure, which could lead to the host rock hydraulic fracturing.

## Introduction

Understanding gas transport processes is one of the key issues in the assessment of radioactive waste repository performance and is the focus of many research programs (e.g., Fate Of Repository GasEs: FORGE, 2009–2013; European Joint Programme on Radioactive Waste: EURAD, 2019–2024). The purpose of these research programs is (i) to develop and calibrate models on experimental set-ups under controlled flow-rate and/or gas pressure injection, and to carry out a series of experiments tests on clay rock samples to study the conditions under which gas breakthrough processes occur, and to analyze rock discontinuities, hydro-mechanical variables, induced desaturation, inflow and outflow gas, and preferential paths created, and (ii) to study upscaling of hydrodynamic properties of the clay material derived from these experiments to develop large spatial scale numerical models of gas migration in the repository.

Many in-situ (boreholes) and laboratory gas breakthrough experiments showed the importance of gas-entry pressure (P_g,e_) in two-phase flow through clay-rich materials (e.g.^1–6^). In laboratory experiments, gas injection pressure is usually applied on one side of a clay sample, where gas pressure is increased until the gas entry pressure is exceeded, and gas ingress in the saturated sample is observed. Once the gas migrates through the clay sample, it changes pore-water pressure, swelling pressure and flowrate observed by the monitoring instruments placed around the sample. When gas breakthrough occurs, gas outflow is recorded from the changes in pressure of the backpressure. Since the clay structure at the point of injection is minimally disturbed, lower injection pressures can be used and hence the risk for unwanted fracturing and reflux is decimated. Due to the lower injection pressures, large injection volumes can be injected what results in a larger radius of influence of the injection.

Constant gas injection rate experiments are usually carried out to induce high gas pressures at the boundary of the clay material to estimate specifically its gas breakthrough pressure. However, the change in gas pressure within the sample is usually abrupt and can lead to the creation of factures especially at the edges of the clay sample (interfaces between the clay and the cell of confinement), and thus to a preferential gas transport through these pathways (fractures). Coupled hydro-gas-mechanical 3D numerical models are difficult to apply to such experiments, that why it is preferred to inject small gas fluxes during longer times to slow down the kinetics of gas migration in the sample and to avoid earlier such mechanical damages in the clay structure.

For example, for the case of the Callovo-Oxfordian (COx) claystone, the rock proposed for hosting the French Deep Geological Repository (DGR) project Cigéo^7^, the measured capillary entry pressure (P_c,e_ = P_g,e_ − P_w_) ranges between 0,3 and 3 MPa for damaged (fissured) COx samples, and can be greater than 5 MPa for intact COx samples^4^.

Two in-situ experiments of gas injection in boreholes inside the Opalines clay have been carried out by Marshall et al.^2^ to characterize gas entry pressure and gas permeability of the Opalines clay. These experiments have been implemented with two events as constant flow rate (“gas threshold pressure test”) and as multi-step constant gas pressure. Simulation of these two-phase flow experiments was based on iTOUGH2 code^8^ with the classical VGM model for hydrodynamic properties of the Opalines clay. However, their analysis was not relevant because neither capillary gas entry pressure P_c,e_ nor hysteresis were modelled.

A recent paper^9^ showed that N_2_ gas pressure inside a chamber, where N_2_ pulses are injected through a borehole inside the COx (PGZ1 experiment,^10–12^), can be highly underestimated when P_c,e_ is neglected in the COx hydraulic properties, especially during long time periods when capillary pressure in the COx becomes higher than its capillary gas entry pressure (P_c_ = P_w_ − P_g_ ≥ P_c,e_). During these periods, the N_2_ gas accumulates more in the chamber, and its pressure increases more significantly due to the high capillary gas entry pressure of the COx. Indeed, the COx behaves as a capillary barrier for gas entry from the chamber deeply in the COx leading to the delay of the desaturation of EDZ and COx, but in a more significative way and in a smallest extend in space during time (by comparison to a simulation by parametrization P_c,e_ = 0). The desaturation of the COx is limited to a thin layer near the EDZ/COx interface, and the kinetic of the diffusion of dissolved N_2_ in the water saturated COx is reduced in time.

In the last past two decades, although many studies dealt with the numerical modelling of gas migration through the different components of a DGR (e.g.^13–15^), none of them studied the impact of a non-zero P_c,e_ in the hydraulic properties of the porous materials representing the host rock, the EDZ, and the engineered barriers materials. The main reason for neglecting P_c,e_ is related to the difficulty of the implementation and verification of the thermodynamic conditions necessary for the switch from single-liquid water to two-phase liquid–gas conditions and vice-versa. Materials in a DGR typically show strong differences in capillary entry pressures. Consideration of P_c,e_ makes it more difficult to achieve a stable and convergent numerical solution of a mathematical problem that involves materials with very contrasting properties.

The purpose of this work is to review the models of gas migration in a DGR at the scale of a HLW disposal cell and of a small repository (“module”) of hundreds of HLW cells in order to assess uncertainties on gas pressure and two-phase flow due to non-negligible P_c,e_. Therefore, we re-simulate the benchmarks models that were proposed in the Euratom 7th Framework Program project FORGE (Fate Of Repository GasEs^14,16,17^) using the modified Van Genuchten–Mualem capillary model VGMPE proposed by^9^ with P_c,e_ ≠ 0 for all materials composing the HLW cells and the drift network. This capillary model has been implemented in new versions of iTOUGH2/EOS5^8^ and TOUGH2-MP^18^ codes by accounting for the thermodynamic changes from single to two-phase conditions with P_c,e_ ≠ 0.

## The studied benchmark models

### The cell scale

**Figure 1:**
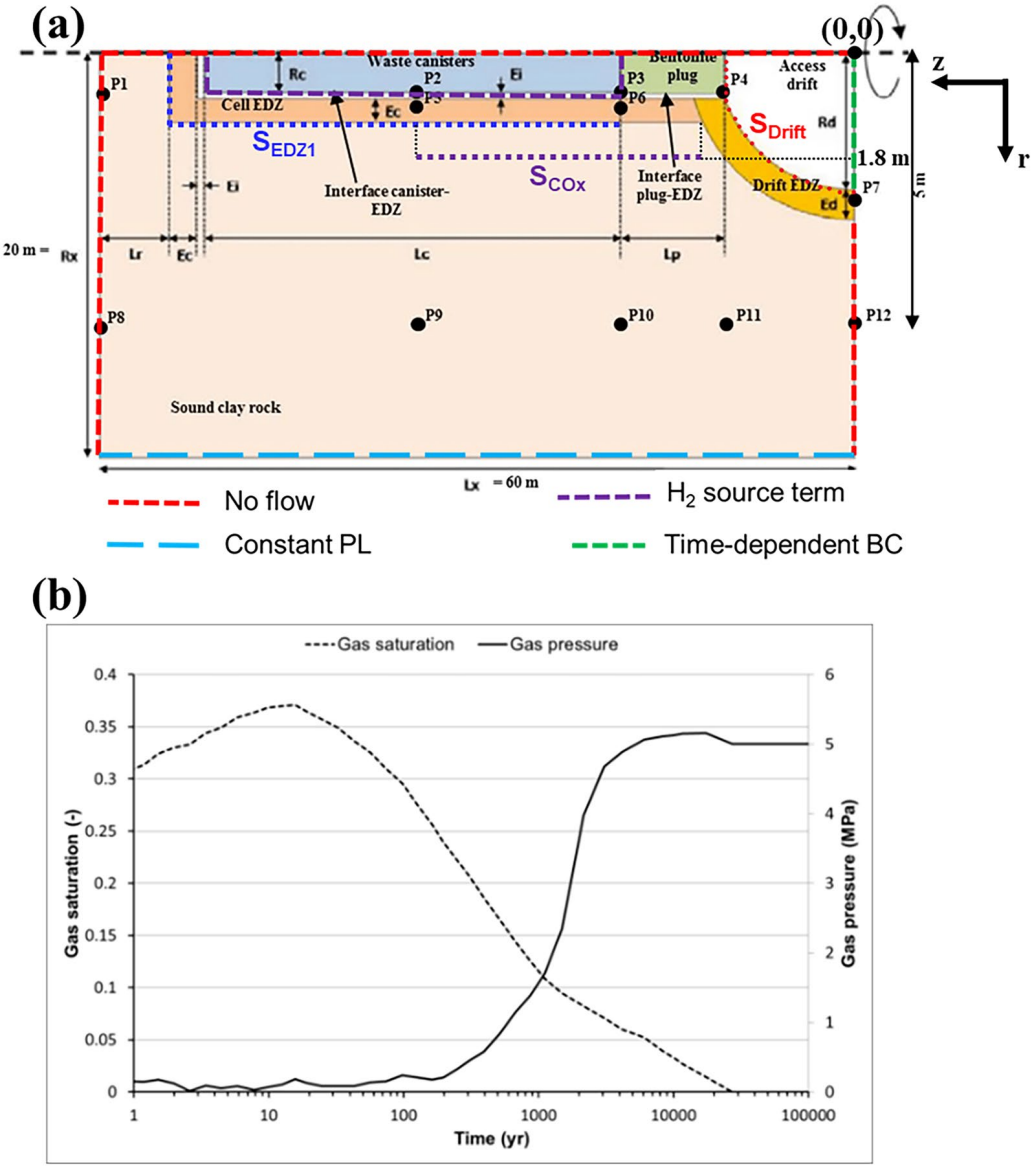
(**a**) Schematic representation of the radial cross section dimensions of the cylindrical waste cell model (assuming plane symmetry along its axial axis), as well as the boundary conditions applied at the surface of the domain. Dark points are those where results should be given (adapted from Wendling et al. 2013a). (**b**) Time-dependent gas saturation and gas pressure applied at the backfilled access drift.

The benchmark model represents an axisymmetric HLW-cell embedded in the COx clay host rock, as shown in Fig. 1a. The interfaces Canisters-EDZ and Plug (bentonite)-COx, considered as a centimeter-thick region, and EDZ around the borehole for canisters emplacement, are also represented. A hydrogen source term due to anoxic corrosion, corresponding to a gas flux rate of 100 mol/year/cell lasting 10 000 years is assumed. It is localized on the waste canister external surfaces (purple continuous line in Fig. 1a).

Gravity effect is assumed negligible compared to capillary forces, because of the very low permeability of all the clay-rich materials composing the cell. Time-dependent boundary conditions are considered in the backfilled access drift (green discontinuous line in Fig. 1a) by assuming time-varying gas pressure and gas saturation, as shown in Fig. 1b ^16^. The simulation time is limited to 100 000 years.

In this work, the classical VGM-model describing the hydraulic properties of the different materials composing the cell has been changed to introduce a non-zero capillary gas entry pressure P_c,e_ (Pa). The new VGMPE model proposed by^9^ has been used. It is a generalization of^19^-model for two-phase flow with a non-zero capillary entry pressure P_c,e_ in the constitutive VGM^20–22^ relationships for the water retention curve ${P}_{c}\left({S}_{l}\right)$ and the relative permeability to liquid and gas curves ${k}_{r,\beta =l,g}\left({S}_{l}\right)$. This model is described by the following equations:

1 $$P_{c} \left( {S_{l} } \right) = \left\{ {\begin{array}{*{20}l} { - \frac{1}{\alpha }\left[ {\left( {S_{e}^{*} S_{e} } \right)^{{ - \frac{1}{m}}} - 1} \right]^{\frac{1}{n}} } \hfill & {\quad if \quad S_{e} \le 1} \hfill \\ 0 \hfill & {\quad elsewhere} \hfill \\ \end{array} } \right.$$

2 $$k_{r,l} \left( {S_{l} } \right) = \frac{{k_{l} \left( {S_{l} } \right)}}{{k_{0,l} }} = \left\{ {\begin{array}{*{20}l} {\left[ {S_{e} } \right]^{\tau } \left[ {\frac{{1 - \left( {1 - \left( {S_{e}^{*} S_{e} } \right)^{\frac{1}{m}} } \right)^{m} }}{{1 - \left( {1 - \left( {S_{e}^{*} } \right)^{\frac{1}{m}} } \right)^{m} }}} \right]^{2} } \hfill & {\quad if \quad S_{e} < 1} \hfill \\ 1 \hfill & {\quad if \quad S_{e} = 1} \hfill \\ \end{array} } \right.$$

3 $$k_{r,g} \left( {S_{l} } \right) = \frac{{k_{g} \left( {S_{l} } \right)}}{{k_{0,g} }} = \left\{ {\begin{array}{*{20}l} {\left[ {1 - S_{e} } \right]^{{\tau^{\prime}}} \left[ {1 - \frac{{1 - \left( {1 - \left( {S_{e}^{*} S_{e} } \right)^{\frac{1}{m}} } \right)^{m} }}{{1 - \left( {1 - \left( {S_{e}^{*} } \right)^{\frac{1}{m}} } \right)^{m} }}} \right]^{2} } \hfill & {\quad if \quad S_{e} < 1} \hfill \\ 0 \hfill & {\quad if \quad S_{e} = 1} \hfill \\ \end{array} } \right.$$

With:

4 $$S_{e}^{*} = \frac{{S_{ls} - S_{lr} }}{{S_{ls}^{*} - S_{lr} }} = \left[ {1 + \left( {\alpha P_{c,e} } \right)^{n} } \right]^{ - m} \quad S_{e} = \frac{{S_{l} - S_{lr} }}{{S_{ls} - S_{lr} }}$$

In the equations above:

$m$ and $n$: are dimensionless shape parameters related by the relation $m=1-\frac{1}{n}$.

$\alpha$ $=\frac{1}{{P}_{r}}$: in (Pa^−1^), with ${P}_{r}$ is a scaling pressure parameter (Pa) ($\alpha$ > 0; if ${P}_{r}$ is positive).

$\tau$ and ${\tau }{\prime}$: are dimensionless parameters representing tortuosity for relative permeability to liquid and gas respectively. In this study $\tau$ = ${\tau }{\prime}$  = 0,5.

${S}_{lr}$ and ${S}_{ls}$: are residual and maximum (or full) liquid saturation, respectively (−). In this study, ${S}_{lr}$ = 0 and ${S}_{ls}$ = 1.

${S}_{e}$: is the effective saturation of liquid phase (−).

${S}_{e}^{*}{S}_{e}$: is the new effective liquid saturation, which reaches its maximal value when ${S}_{l}={S}_{ls}=1$ (−).

${k}_{0,l}$ and ${k}_{0,g}$ : are scaling permeability parameters, defined as intrinsic permeabilities to liquid and gas, respectively (m^2^).

In Eq. (1) the water retention curve is extended beyond the full liquid saturation ($${S}_{ls}=1)$$ by introducing the parameter $${S}_{ls}^{*}$$ ≥ 1 in the capillary VGM model. As reported in^9^, relative permeability to gas and liquid is more sensitive to P_c,e_ than the water retention curve near full liquid saturation. Notice that when P_c,e_ = 0, Eq. (4) implies that $${S}_{e}^{*}=1$$ and $${S}_{ls}^{*}={S}_{ls}$$, then Eqs. (1), (2) and (3) are reduced to the classical VGM-model equations proposed in^22^.

The physical properties (i.e., porosity, intrinsic permeability, pore compressibility…) of the different materials composing the disposal cell, and parameters of their hydrodynamic properties in Eqs. (1), (2) and (3), are given in Table 1^16^. The P_c,e_-value 2 MPa for the COx has been optimized through best fit of P_c_(S_l_), k_r,l_(P_c_), k_r,g_(P_c_) data and has been used for the PGZ1-experiment model^9^. The P_c,e_-values of bentonite and EDZ are deduced in % of COx P_c,e_-value optimized (2 MPa) by analogy to those measured on average for the three materials. This % is evaluated independently, from the ratio of measured P_c,e_-values of bentonite and EDZ (~ 4 and 2 MPa, respectively;^23,24^) to measured P_c,e_-value of the COx (~ 6 MPa;^23,24^). Therefore, P_c,e_-values of bentonite and EDZ are equal to 4/6 (67%) and 2/6 (33%) times 2 MPa, respectively (Table 1). The P_c,e_-value 0.1 MPa for the backfill is an approximation of the value 0.09875 MPa which has been evaluated through re-fitting simultaneously P_c_(S_l_), k_r,l_(P_c_), and k_r,g_(P_c_) numerical data calculated by Eqs. (1), (2) and (3) with P_c,e_ = 0. This fitting, using the optimization algorithms developed by^9^, did not highly modify values of the shape parameter ‘*m*’ and the scale parameter ‘*P*_*r*_’ in Eqs. (1), (2) and (3), given in Table 1. Like the EDZ material, the backfill has a porous structure composed of clay blocks with high capillarity (high P_c,e_), and big inter-block voids without capillarity (P_c,e_ = 0). Although P_r_-value of the backfill is higher than that of the EDZ, its P_c,e_ value is smaller than that of the EDZ. This is in accordance with its higher permeability and porosity compared to those of the EDZ (Table 1).

**Table 1:** Physical properties and parameters of VGMPE hydrodynamic properties of the materials used in the simulations (^16,17^).

Material	Porosity (−)	Intrinsic permeability (m^2^)		Pore compressibility (Pa^−1^)	Capillary entry pressure, *P*_*c,e*_ (Pa)	VG pressure scale parameter, *P*_*r*_ (Pa)	VG shape parameter, *n* (−)
		Waste cell scale^a^	Module scale				
Geological Medium (clay host rock)	0.15	7.94 × 10^–21^	1.0 × 10^–20^	1.11 × 10^−9^	2.00 × 10^6^	1.5 × 10^7^	1.5
EDZ	0.15	7.94 × 10^–18^	5.0 × 10^–18^	1.11 × 10^−9^	6.67 × 10^5^	1.5 × 10^6^	1.5
Bentonite	0.35	1.0 × 10^–20^	1.0 × 10^–20^	8.25 × 10^−10^	1.33 × 10^6^	1.6 × 10^7^	1.6
Backfill	0.40	5.0 × 10^–17^	5.0 × 10^–17^	2.09 × 10^−9^	1.00 × 10^5^	2.0 × 10^6^	1.5
Interface facing plug	0.30	7.94 × 10^–18^	5.0 × 10^–18^	1.11 × 10^−9^	0	1.0 × 10^4^	4
Interface facing canister	1.00	1.0 × 10^–12^	1.0 × 10^–12^	1.09 × 10^−11^	0	1.0 × 10^4^	4
Interface facing backfill	0.40	1.0 × 10^–15^	1.0 × 10^–15^	2.09 × 10^−9^	0	1.0 × 10^4^	4

^a^Radial intrinsic permeability. Calculated from horizontal and vertical intrinsic permeability values using geometric mean: (K_x_ × K_y_ × K_z_)^1/3^.

As shown in Table 1, interface facing canister is constituted by voids (porosity of 100%, like a fracture with a thin layer between two parallel plates, whose permeability is calculated by Poiseuille law for the fluid flow). The interface facing bentonite plug is a mixture of big voids with the porous materials bentonite and EDZ (COx matrix and fractures), but it is largely made up of voids with important roughness. This explains its higher permeability, which is 100 times greater than that of bentonite and close to that of the EDZ. Interface facing backfill differs from that facing the canister by a different mixture of backfill, EDZ, and big voids. Its permeability is higher than the interface facing the plug because of the high permeability of the backfill compared to that of bentonite in the mixture, and to a small roughness. The three interfaces are largely made up of voids and flow by capillarity in their porous structures is negligible. Their scale parameter P_r_ in VGMPE model is chosen very small (Table 1), and therefore have negligible P_c,e_ (P_c,e_ ≈ 0).

Transport properties are such that: the diffusion coefficients of water vapor and hydrogen in unsaturated porous materials are deduced from their diffusion coefficients in free gas and liquid through the proportionality constant (tortuosity factor) calculated by the Millington and Quirk formula^25^. Diffusion coefficients of hydrogen in free liquid and gas phases are equal to 6.0 × 10^–9^ and 9.5 × 10^–5^ m^2^/s^17^, respectively, and the diffusion coefficient of water vapor in free gas phase is equal to 2.1 × 10^–5^ m^2^/s^15^. The inverse Henry’s constant of hydrogen is equal to 1.379 × 10^–10^ Pa^−1^.

The initial gas saturation is 30% for bentonite seals (cell and main drift) and for backfill (drifts). An initial gas saturation of 95% for all interfaces is considered. For these materials initially unsaturated, the gas pressure is equal to 1 atmosphere (~ 1 bar = 0.1 MPa), and the water pressure is deduced from the gas pressure and water saturation. Therefore, liquid pressure in capillary equilibrium with gas pressure is deduced from gas pressure and liquid saturation by applying the VGM-model of the water retention curve defined by Eq. (1). The clay host rock, and cell and drift EDZs are initially at full liquid saturation with an initial water pressure set to 5 MPa.

The mesh of the domain used for the simulations is like that recently presented in^15^. It is radial and rectilinear; it consists of 39 × 122 = 4758 elements in the plane (r, z). This number is reduced to 4358 active elements because elements representing the waste canister are not considered in the calculation. Two extra-cells with infinite volumes were added to handle boundary conditions (BC). The first one is for fixing the time-dependent gas pressure PG and gas saturation SG (Fig. 1b) at the drift exit boundary Z = 0 (green discontinuous line in Fig. 1a), and the second one is for fixing a constant liquid pressure PL of 5 MPa at r = 20 m (blue discontinuous line in Fig. 1a).

The purpose is to follow during 100 000 y the state variables gas saturation (SG), and gas and liquid pressures (PG, PL) at points P1-P18, as well as convective and diffusive fluxes of H_2_ in liquid and gas phases at sections S_EDZ1_, S_drift_ and S_COx_, as shown in Fig. 1a.

### The repository scale “module”

In this subsection, we aim to develop a 3D model of H_2_ transport at the scale of a small repository (module) of many HLW disposal cells (which is only part of a repository of several parallel drifts and modules) by simulating simultaneously all H_2_ sources (located in cells), and H_2_ transfer pathways in the underground drifts network (access and main drifts) during the post-closure phase of a DGR, i.e. for 100 000 y. The studied module is that presented and documented in FORGE benchmark^14,17^.

The purpose of the study of gas migration within this module is two-fold:

(i) to verify our numerical results simulated for a parametrization P_c,e_ = 0 by confronting them to those obtained by other teams that participated to this benchmark. This step is essential for the credibility of our model, which we intend to use in a future work to simulate gas migration scenarios at the scale of a DGR composed of several modules. These scenarios could include different physical sub-models and associated parameters representing the source term of H_2_ and its solubility, and thermo-hydraulic-gas properties of the clay host rock, EDZ, engineered barriers materials (backfill and bentonite-plug), and interfaces between EDZ and these materials.
(ii) To investigate the impact of the new parameterization P_c,e_ ≠ 0 (for all materials) on gas pressure and water–gas fluxes within the module by using the new VGMPE model for hydraulic properties (Eqs. (1)–(4)). This can help to draw new conclusions about the real situation of gas entry within the host rock and the engineered barriers materials.

The main difficulty in this study is to simulate the physical model with less CPU time. Therefore, the mathematical problem should describe properly the physics of two-phase flow in the porous materials composing the module (disposal waste cell and drifts network). The “conceptual model” includes mathematical descriptions of physical processes, as well as model geometry, and spatial distribution of materials with different properties, as well as their spatial discretization (mesh).

#### The modelled domain

**Figure 2:**
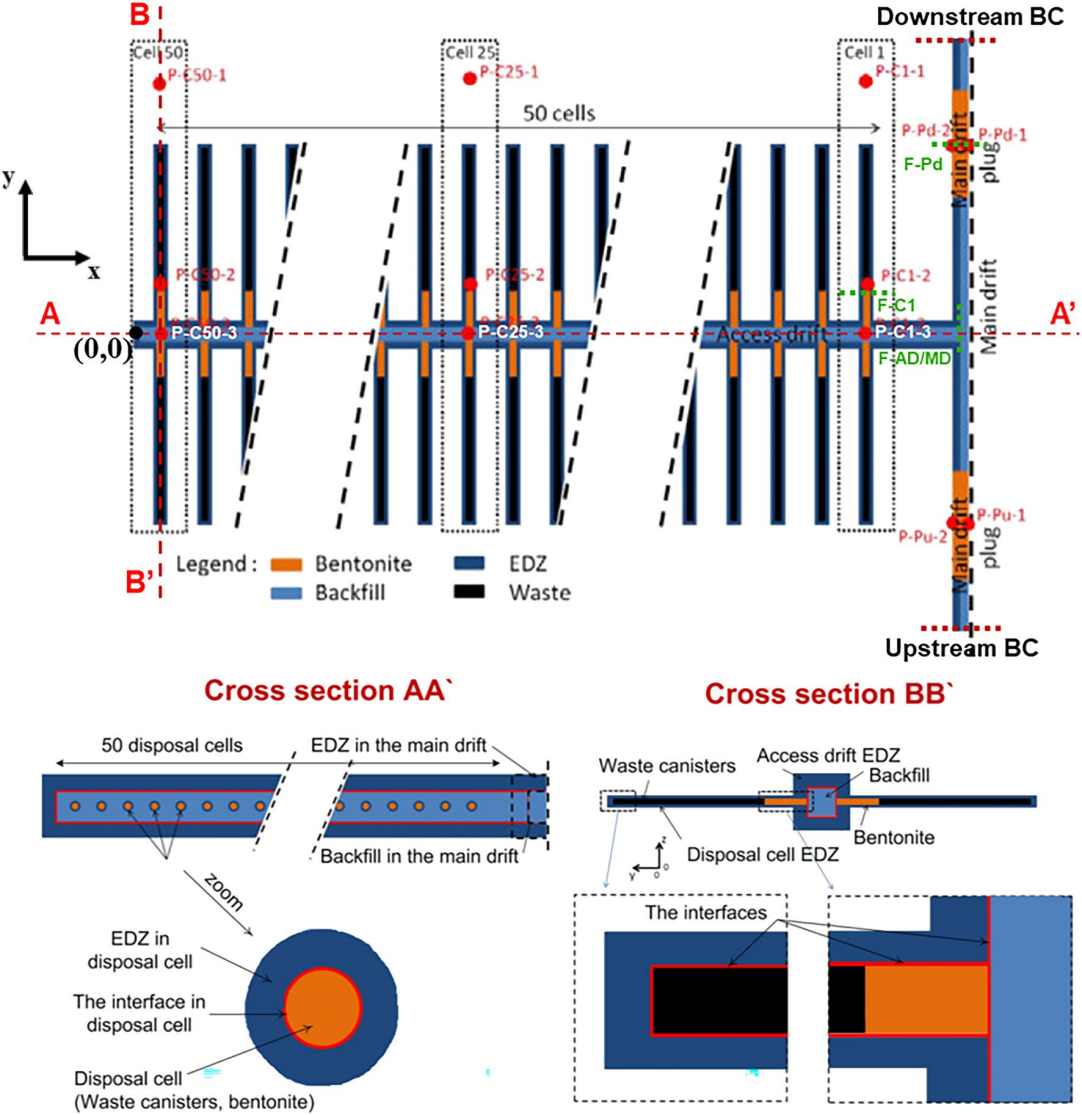
Above: 2D view of the module in the plane XY at Z = 75 m. Below: 2D view in the XZ plane (cross section AA’) of the main drift, and 2D view in the YZ plane (cross section BB’) of the access drift and of the disposal waste cell. Access and main drifts have the same squared cross Sect. (6 × 6 m^2^). The access drift is completely backfilled (Maya blue color). The main drift is partly backfilled: presence of a bentonite seal with a cross section of 8 × 8 m^2^ in the plane XZ (orange color). Interfaces of 1 cm thickness are shown by red lines (Adapted from Wendling et al.^17^).

**Figure 3:**
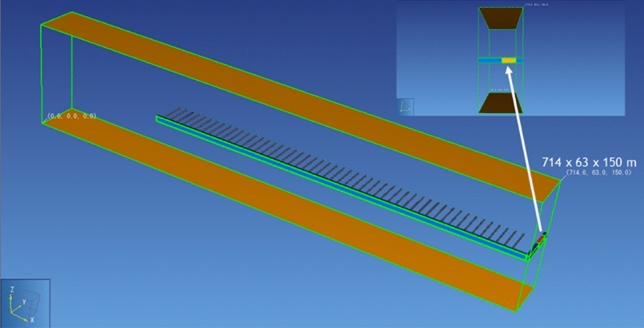
The 3D view of the module embedded in the COx (preprocessing under PetraSim).

The studied module is in the middle of a raw of 5 modules along Y-axis (having the same geometry) in the whole repository (10 modules, with a raw of 5 modules at each side of the main drift; see Supplementary Fig. S1 online). It consists of 50 cells at each side of the access drift (Fig. 2). Because of the module position within the repository, time-dependent gas pressure PG(t) and gas saturation SG(t) at downstream and upstream BCs (Fig. 2 above) are very similar. These time-dependent PG(t) and gas saturation SG(t), like those shown in Fig. 1b, were calculated using the full 3D repository model. Therefore, the fact that the central XZ-plane of the access drift is a symmetry axis for the disposal cells, only the downstream BC has been modelled (Fig. 2). So, the size of the modelled domain is reduced to the volume 714 × 63 × 150 m along X, Y, and Z-axis, respectively. Figure 3 shows a 3D view of the modelled geometry of the module which has been embedded within the COx. Each HLW disposal cell shown in Fig. 2 has the same characteristics as described in Fig. 1a. The vertical extend of the domain is limited to the layer thickness of COx (150 m). The horizontal extend in the other directions (OX, OY) is representative to the distance between cells (10 m). The porous materials considered in the model are the backfill in drifts, bentonite for plugs (seals) in cells and main drift, the COx clay host rock, the EDZ, and interfaces EDZ-backfill, EDZ-bentonite and COx-bentonite. The so-called “cell” consists of the waste canisters and the bentonite plug of the micro-tunnel (Fig. 1a).

Geometries of the disposal cells and drifts composing the module within the COx are detailed in Wendling et al.^17^. However, some approximations have been considered in our simulations:

- Each circular cross section of the disposal cell has been equated to a square cross section (same area) in order to simplify introduction of disposal cells centimetric interfaces in a rectilinear mesh. This approximation is the same as that adopted by other teams that contributed to FORGE benchmark (e.g.^26,32^).
- The waste canisters are not modelled and assumed impermeable to water and gas. The elements that represent the canisters are excluded from the calculations. The boundaries to neighboring elements are no-flux boundaries. A constant hydrogen source term of 100 mol/y/cell lasting 10 000 y is attributed to each disposal cell, it is applied at the waste cylinder surface.
- To avoid the severe numerical convergence problems caused by the infinitesimal volume of the centimetric interfaces located near coarser mesh elements, the space-discretization near interfaces is simplified by homogenizing them with a fraction of one of their adjacent materials, as proposed by^26^ and applied by^27^. In fact, the combination interface-adjacent material has been carried out as follows: for cells, the interfaces EDZ-canister and EDZ-bentonite seal are combined with 0.24 m of EDZ; for drifts, the EDZ-backfill is combined with 0.49 m of backfill while the COx-bentonite interface is combined with 0.49 m of bentonite. This approach leads to changing transport and hydraulic properties, and initial conditions of the homogenized layer. A recent analysis^14^ showed that differences between results simulated with and without homogenization of interfaces in the mesh were not substantial.

#### The model implementation

Initial and boundary conditions of the model have been implemented as described in the benchmark specifications^17^:

- A Newman type BC with zero-flux is imposed at the lateral boundaries, except at the exit boundary of the main drift, where a Dirichlet BC is imposed with time-dependent gas saturation and gas pressure (idem curves in Fig. 1a).
- At the bottom (Z = 0) and top (Z = 150 m) boundaries, Dirichlet BCs are imposed with constant hydrostatic pressures of 4 and 6 MPa, respectively.
- The initial unsaturated conditions in the materials composing the module are the same as those composing the disposal waste cell. However, for the saturated materials initially at full water saturation, clay host rock and EDZs, the initial water pressure is not constant but calculated from hydrostatic equilibrium between Z = 0 and Z = 150 m before drifts excavation.

The physical properties, VGMPE-hydrodynamic parameters (Eqs. (1)–(4)), and the two-phase hydrogen diffusion coefficients of the different materials composing the module are the same as those composing the disposal cell (subsection “The cell scale”, Table 1).

**Figure 4:**
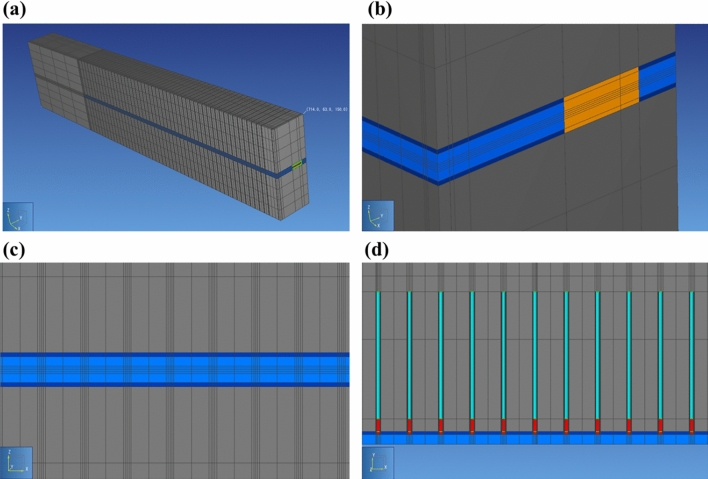
(**a**) Mesh of the studied domain. (**b**) Zoom on the drifts mesh. (**c**) Mesh of the access drift in plane XZ. (**d**) Mesh near cells in plane XY.

Pre-processing of the model mesh and its visualization are carried out via PetraSim^28^, as shown in Fig. 4a. Figure 4b,c,d show a zoom on the mesh over all the materials which constitute the disposal cells and drifts of the module within the COx.

The mesh of the module is also rectilinear, as for the cell mesh. It is composed of about 412 × 11 × 21 = 95 172 elements (including 150 elements representing the mesh of waste canisters that were deactivated, not used for the calculations). Three extra-elements with infinite volumes are added for handling Dirichlet BC’s, two of which for imposing constant hydrostatic liquid pressure at bottom and top vertical boundaries (Z = 0, 150 m), and one of which for imposing time-dependent gas pressure and gas saturation (Fig. 1b) at the main drift exit (Y = 63 m).

In this study, the state variables gas saturation (SG), and gas and liquid pressure (PG, PL) are followed during 100 000 y at points elements located near cells C1, C25 and C50, and the two points elements P-Pd-1 et P-Pd-2 located in the bentonite-plug of the main drift, Fig. 2. The points elements P-C1-1, P-C25-1, P-C50-1 are situated in the COx; the points elements P-C1-2, P-C25-2, P-C50-2 are situated in the homogenized EDZ-interface (upper part) connected to the bentonite plug; and the points elements P-C1-3, P-C25-3, P-C50-3 are situated in the backfilled access drift. In addition, convective and diffusive H_2_ fluxes in liquid and gas phases at sections FC-1, F-AD/MD and F-Pd (green, dashed lines in Fig. 2) are also followed during 100 000 y. F-C1: vertical plane passing through the end (close to the waste) of the cell bentonite plug, cell EDZ, and homogenized layer interface-cell EDZ; F-AD/MD: vertical plane at the access drift-main drift interface, passing through backfill, drift EDZ, homogenized layer interface-drift EDZ; and F-Pd: vertical plane passing through the middle of the downstream main drift plug and homogenized layer interface-bentonite.

### The numerical solution

Simulations are performed using the TOUGH-suite of codes iTOUGH2^8^ and TOUGH2-MP^18^. Both codes have been used to solve many numerical problems of non-isothermal two-phase flow and two components (water-hydrogen) transport in both liquid and gas phases in porous media, especially hydrogen migration at different scales of a DGR (e.g.^14,15^). The advantage of using the massively parallel code TOUGH2-MP is that it uses the domain decomposition method, which is designed for parallel simulation in multi-CPU platforms (multicores processing). The equation of state 5 (EOS5)^29^ has been chosen to describe thermodynamics of H_2_ gas in presence of water.

The numerical solution for each of the two specified models presented above is based on a modified version of TOUGH2-MP/EOS5 by including the VGMPE model as implemented in iTOUGH2/EOS5^9^. This modification led to the development of two numerical approaches:

- The P_c_-method (PCM) which consists of changing the primary variable S_g_ by the capillary pressure variable P_c_ for the switch from single-phase liquid to two-phase liquid–gas conditions, and vice-versa.
- The S_g_-method (SGM), which introduces thermodynamic conditions for this transition.

Both numerical methods give accurate results by comparison (benchmarking) to an analytical solution and two numerical codes for single-phase liquid unsaturated flow^9^. Moreover, solutions of both numerical schemes were in a good agreement with N_2_-gas pressure measured in a borehole of the COx during periods of six pulses of N_2_ injection in the PGZ1-experiment carried out at the URL of Bure^10–12^. As shown in^30^, the numerical scheme SGM is better in terms of CPU-time for PGZ1-experiment simulation by comparison to PCM scheme, because it drastically reduces the number of Newton–Raphson iterations and time steps when P_c,e_ ≠ 0, and this for both coarse and fine meshes tested. Therefore, the SGM numerical scheme has been chosen for this study.

## Results and discussions

Because of isothermal conditions at 20 °C, the water vapor partial pressure is constant in time and equal to 2337 Pa. This value is very small compared to that of H_2_ (between 0.1 and 9 MPa). Idem, the water vapor flux is very negligible compared to that of H_2_. Therefore, in what follows, the gas phase is essentially composed of hydrogen.

### The cell scale: P_c,e_ = 0 vs P_c,e_ ≠ 0 simulations

**Figure 5:**
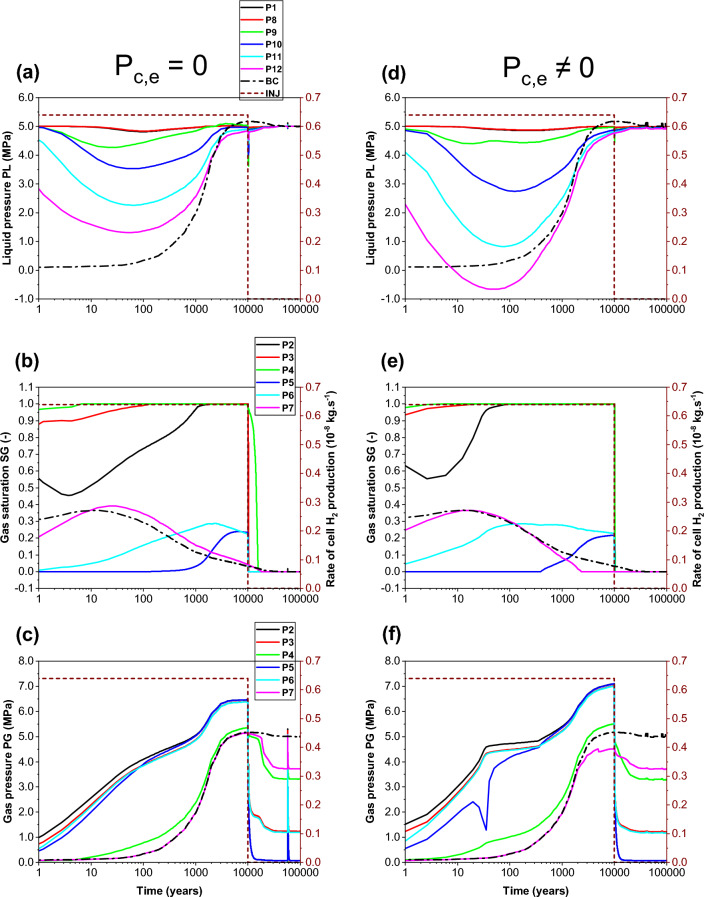
Evolutions in time of state variables simulated by SGM numerical scheme and the VGMPE model for hydraulic properties. (**a**, **d**) Liquid pressure at points P1 and P8-P12; (**b**, **e**) gas saturation degree at points P2-P7; (**c**, **f**) gas pressure at points P2-P7. (**a**, **b**, **c**) Parametrization P_c,e_ = 0 (left); (**d**, **e**, **f**) Parametrization P_c,e_ ≠ 0 (right). BC: boundary condition; INJ: injected mass of hydrogen per unit time, generated by the corrosion of canisters of the disposal waste cell during 10 000 y.

From the comparison of time-varying liquid and gas pressure (PL and PG), and gas saturation (SG) simulated at points elements P1–P12 (Fig. 1a) during 100 000 y by both parametrizations, P_c,e_ = 0 (Fig. 5a,b,c) and P_c,e_ ≠ 0 (Fig. 5d,e,f), we can conclude :

- COx: although points P1 and P8–12 remain saturated during the whole simulation period (100 000 y), their liquid pressure PL decreases more during transient phases for the parametrization P_c,e_ ≠ 0 (Fig. 5d). This is essentially due to the increase of dissolved H_2_ mass generated by canisters metal corrosion that is diffusing in liquid water towards points P1 and P8–12. The gas flow from EDZ (lower gas entry pressure) into intact COx (higher gas entry pressure) is only possible if gas pressure is above gas entry pressure of the COx (development of 2-phase liquid–gas thin zone in the near EDZ-COx interface). This leads to a temporary blockage of H_2_ gas at the EDZ-COx interface. The point P12 is much more impacted by the substantial dissolved H_2_ mass (negative PL during the period ~ 10–240 y) due to its presence near the access drift BC (source of H_2_, Fig. 1b).
- Interfaces: a fast displacement of gas near the access drift at points P3 and P4 is simulated for a parametrization P_c,e_ ≠ 0 (Fig. 5e); the gas saturation SG quickly reaches 1.0. Far from the access drift (point P2), SG reaches 1.0 later after ~ 100 y (~ 2000 y for the parametrization P_c,e_ = 0).
- EDZ: for the parametrization P_c,e_ ≠ 0, there is a significant and fast water-desaturation of the EDZ in the nearest points to the access drift (points P6, P7); there is also a late arrival of H_2_ at the farthest point to the access drift, P5 (~ 400 y, Fig. 5e).
- For points P2 and P3 in the interface and points P5 and P6 in the EDZ located far from the access-drift, the gas pressure increases by about 0.6 MPa at 10 000 y (from 6.47 to 7.1 MPa) for a parametrization P_c,e_ ≠ 0 (Fig. 5f). This is due to an important accumulation of H_2_ at interfaces and backfill of the access drift for this parametrization.

**Figure 6:**
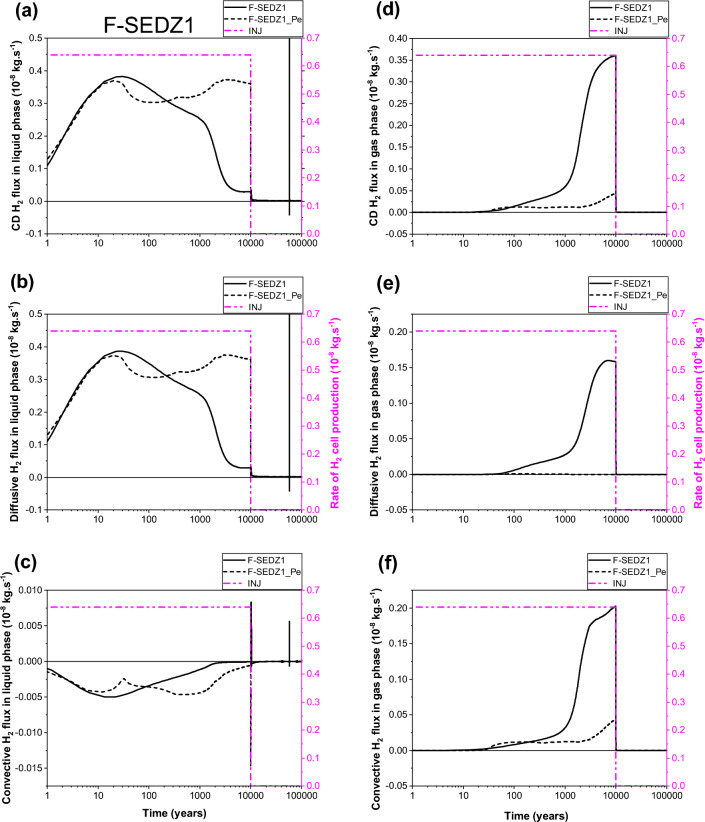
Evolutions in time of H_2_ flux simulated at cross section S_EDZ1_ (Fig. 1) by using both parametrizations P_c,e_ = 0 and P_c,e_ ≠ 0. (**a**, **d**) Convection–Diffusion (CD); (**b**, **e**) Diffusion (D); (**c**, **f**) Convection (C). (**a**, **b**, **c**) Liquid phase; (**d**, **e**, **f**) Gas phase. INJ: injected mass of hydrogen per unit time, generated by the corrosion of canisters of the disposal waste cell during 10 000 y.

Looking at Fig. 6a,b,c, one can see, for both parametrizations (P_c,e_ = 0 and P_c,e_ ≠ 0), that the transport of dissolved H_2_ in liquid–water phase through section SEDZ1 (Fig. 1a) is mainly by diffusion from the EDZ to the clay host rock (COx). Both parametrizations simulate a small convective flux for the transport of dissolved H_2_ in liquid phase from the COx towards the EDZ, but with an important delay of ~ 6000 y for parametrization P_c,e_ ≠ 0 before reaching negligible value after 10 000 y. This convective flux is essentially produced by a capillary pressure gradient between the COx (rich of dissolved hydrogen due to diffusion in liquid phase), and interface canister-EDZ which dries faster (liquid saturation of 0%) during the gas production period in the case of parametrization P_c,e_ ≠ 0 (see also points P2, P3, P4 in Fig. 5e).

**Figure 7:**
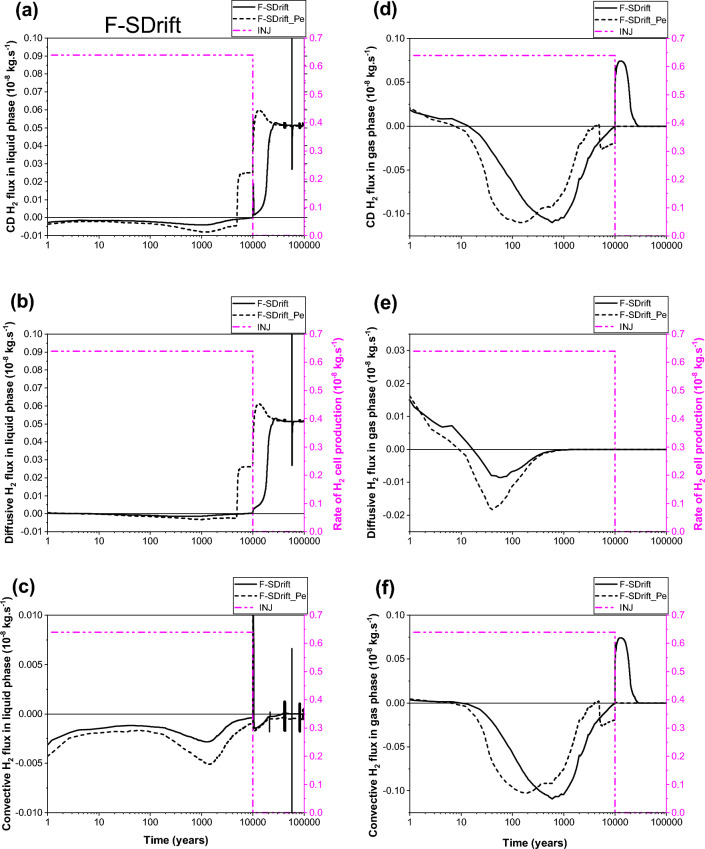
Evolutions in time of H_2_ flux simulated at cross section S_Drift_ (Fig. 1) by using both parametrizations P_c,e_ = 0 and P_c,e_ ≠ 0. (**a**, **d**) Convection–Diffusion (CD); (**b**, **e**) Diffusion (D); (**c**, **f**) Convection (C). (**a**, **b**, **c**) Liquid phase; (**d**, **e**, **f**) Gas phase. INJ: injected mass of hydrogen per unit time, generated by the corrosion of canisters of the disposal waste cell during 10 000 y.

**Figure 8:**
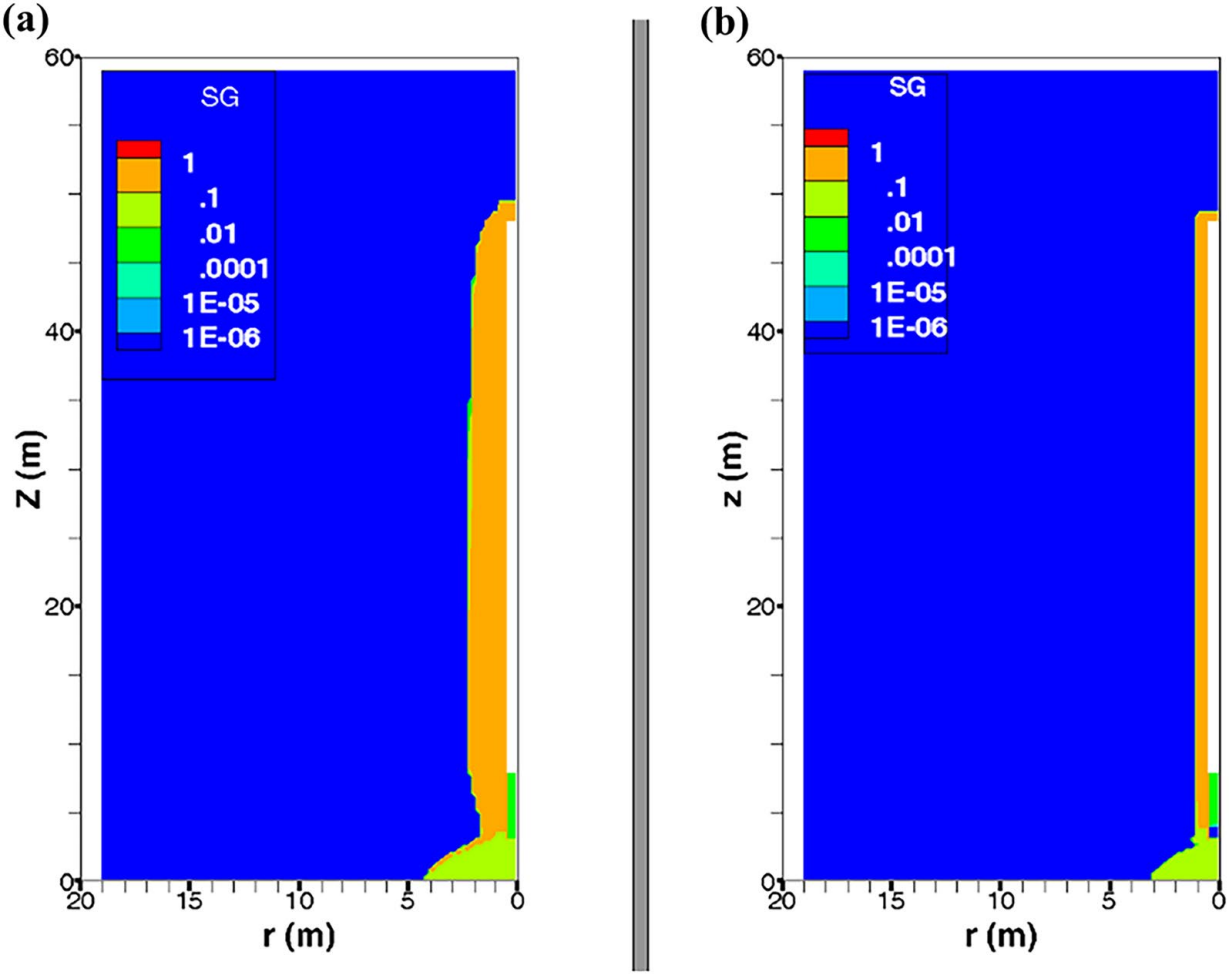
2D profiles of gas saturation at time t = 10 000 y simulated by our model, by iTOUGH2/EOS5 (numerical scheme SGM) and VGMPE-model for hydraulic properties: (**a**) Parametrization P_c,e_ = 0; (**b**) Parametrization P_c,e_ ≠ 0.

The most surprising result is that shown in Fig. 6a,b, the diffusion of dissolved H_2_ in liquid–water towards the COx remains higher during all the period of gas production (10 000 y) when parametrization P_c,e_ ≠ 0 is considered. This is essentially due to negligible desaturation of the COx around the EDZ, and the very small diffusive-convective flux of H_2_ in the gas phase towards the COx (short, dashed lines in Fig. 6d,e,f). However, for parametrization P_c,e_ = 0, both diffusion and convection processes in the gas phase towards the EDZ are substantial (solid lines in Fig. 6e, f). Because of the high P_c,e_-value of the COx, H_2_ in the gas phase of EDZ cannot deeply penetrate the COx only if the difference between gas pressure in the EDZ and liquid pressure in the COx is greater than the capillary entry pressure of the COx, P_c,e_ = 2 MPa. Therefore, H_2_ migrates fast through the gas phase of centimetric interfaces and EDZ with low P_c,e_-values towards the backfilled access drift, as shown by the convection dominant transport of H_2_ at section SDrift in Fig. 7d,e,f, during the first ~ 300 y (short, dashed lines, parametrization P_c,e_ ≠ 0). Beyond ~ 300 y, the convective H_2_ flux in gas phase simulated by both parametrizations (P_c,e_ = 0 and P_c,e_ ≠ 0) is reduced due to the increase of water saturation in the access drift according to the time-dependent gas saturation (Fig. 1b) applied at the access drift boundary (Fig. 1a). Notice, however, that such a reduction is faster for simulations by parametrization P_c,e_ ≠ 0. For this parametrization, the convective H_2_ flux in gas phase vanishes drastically once the gas production stops at time 10 000 y, whereas a reversible convective H_2_ flux in the gas phase from the access drift to the drift EDZ for parametrization P_c,e_ = 0 is simulated for a period of about 20 000 y until the access drift becomes fully saturated at ~ 30 000 y (corresponding to a gas saturation of ~ 0% in Fig. 1b). This explains the extend of the unsaturated zone in the COx at a radial distance of about 2 m around the EDZ at 10 000 y, as shown in Fig. 8a. For both parametrizations (P_c,e_ = 0 and P_c,e_ ≠ 0), there is a small convective transport of dissolved H_2_ in liquid–water towards the access drift (section SDrift) during almost the whole simulation period. At ~ 5000 y the transport becomes diffusion-dominated towards drift EDZ, interface plug-EDZ and the plug for parametrization P_c,e_ ≠ 0, and later at ~ 10,000 y for parametrization P_c,e_ = 0 (delay of about 5000 y). Notice that the diffusion process in liquid phase is more important for parametrization P_c,e_ ≠ 0 due to H_2_ confinement in interfaces and EDZ, as also shown in Fig. 6b for the diffusive flux at SEDZ1. Both parameterizations simulate a constant diffusion in liquid phase once the access drift becomes fully saturated at ~ 30 000 y (Fig. 1b).

**Figure 9:**
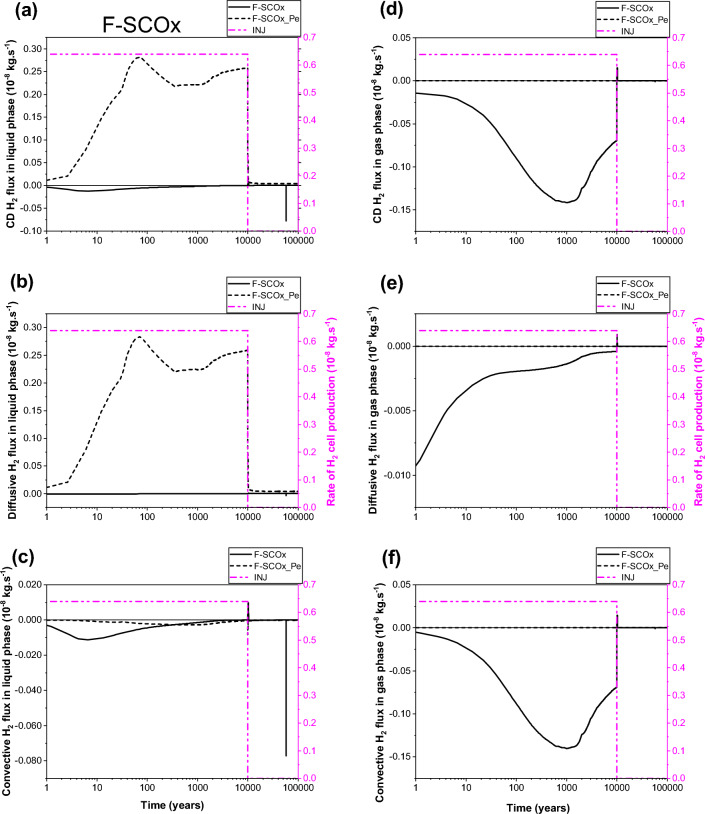
Evolutions in time of H_2_ flux simulated at cross section S_COx_ (Fig. 1) by using both parametrizations P_c,e_ = 0 and P_c,e_ ≠ 0. (**a**, **d**) Convection–Diffusion (CD); (**b**, **e**) Diffusion (D); (**c**, **f**) Convection (C). (**a**, **b**, **c**) Liquid phase; (**d**, **e**, **f**) Gas phase. INJ: injected mass of hydrogen per unit time, generated by the corrosion of canisters of the disposal waste cell during 10 000 y.

The flux of dissolved H_2_ in liquid phase and that of H_2_ in the gas phase simulated at section SCOx at a radial distance r = 1.8 m (0.8 m away from the waste cell EDZ-COx interface, Fig. 1a), shown in Fig. 9, confirm again simulations observed at section SEDZ1 (Fig. 6). For parametrization P_c,e_ ≠ 0, the fact that neither diffusion nor convection of H_2_ in the gas phase are responsible for hydrogen migration to SCOx (Fig. 9d,e,f), all the hydrogen reaching SCOx is that transported by diffusion-only of dissolved H_2_ in liquid–water from EDZs to the saturated COx, Fig. 9a,b,c. Therefore, section SCOx remains saturated, as also confirmed by gas saturation profile at 10 000 y shown in Fig. 8b. However, for the parametrization P_c,e_ = 0, a convection-dominated transport in the gas phase is simulated at section SCOx, from COx towards EDZs and interfaces (cell EDZ—canister, cell EDZ—plug), showing that a two-phase flow is developed from SCOx to the EDZ/COx interface all over the period of gas production 10 000 y (see gas saturation profile at 10 000 y in Fig. 8a, where SCOx at r = 1.8 m is unsaturated, as well as solid line for convective-diffusive flux at section SDrift in Fig. 7d). This convective flux is reduced in parallel to the increase of water saturation in the access drift, EDZs and interfaces. Also notice the rapid cancellation of flow once gas production stops at 10 000 y for this parametrization (P_c,e_ = 0).

Some numerical oscillations that are non-physical appear in the calculation of small liquid fluxes after 10 000 y, especially when parametrization P_c,e_ = 0 is considered (whether by convection or by diffusion) in all Figs. 6, 7 and 9. These oscillations, due to numerical dispersion, disappear almost totally for parametrization P_c,e_ ≠ 0. Vogel et al.^19^ made the same observations in their numerical simulations of an experiment of capillary rise of water in a 1D clay column of 1 m length by solving Richards’ equation. Notice, however, the drastic change of convective transport of H_2_ in liquid phase for parametrization P_c,e_ ≠ 0 near discontinuity in time at 10 000 y due to small changes in the time step.

To summarize, from analysis of 2D-profiles of gas saturation at 10 000 y (Fig. 8), time-evolution of PL, PG and SG in points P1–P12 (Fig. 5), and diffusive and convective fluxes of H_2_ in liquid and gas phases through sections SEDZ1, SDrift and SCOx (Figs. 6, 7 and 9), we can conclude that:

- For a parametrization P_c,e_ = 0, there is simulation of two-phase flow pathways showing an important advance of H_2_ front in the COx in the radial direction of the disposal cell, leading to development of an unsaturated zone around EDZs (Fig. 8a). This zone is especially due to important H_2_ diffusion and convection in liquid and gas phase (supported by Figs. 6, 7 and 9).
- For parametrization P_c,e_ ≠ 0, the advance of gas front in the COx is limited to the near EDZ/COx interface (Fig. 8b). However, there is simulation of an important dissolved H_2_ front in the saturated COx due to diffusion-only from the EDZs to the COx as shown in Figs. 6 and 9.
- For parametrization P_c,e_ ≠ 0, there is a fast water re-saturation of the access drift and of the thin unsaturated zone developed near the disposal cell. This is the consequence of the discontinuity in the VGMPE model hydraulic properties near full water saturation (Eqs. (1), (2) and (3)) that are well implemented in the SGM-scheme of TOUGH2/EOS5 code.
- Finally, for parametrization P_c,e_ ≠ 0, the bentonite-plug begins to re-saturate earlier, which explains also the abrupt decrease of S_g_ from 1 to 0 in point P4 (Fig. 5e).

For the given H_2_ generation rate produced by corrosion of waste canisters in the disposal cell during 10 000 y, it is clear from simulations by parametrization P_c,e_ ≠ 0 that the COx remains saturated and behaves as a capillary barrier. Therefore, H_2_ cannot deeply migrate in the COx through capillary two-phase flow pathways by convection-dominated transport as long as the difference between gas pressure in the EDZ and liquid–water pressure in the COx is less than the P_c,e_-value of the COx.. This is due to the highest P_c,e_-value of the COx with regards to the other materials in the modelled domain (Table 1). Because the COx desaturates only in the near interface EDZ-COx, H_2_ remains confined in the interfaces and EDZ, and therefore preferentially migrates only by diffusion-dominated transport in liquid phase deeply in the COx, and by convection-dominated transport in gas phase towards the access drift.

Moreover, on the one hand there is a fast water drainage in the materials with the lowest P_c,e_-values (interfaces, EDZ, backfilled access drift) during the gas accumulation in these materials; on the other hand, there is a quasi-instantaneous re-saturation of these materials after the period of H_2_ production (10 000 y). These results confirm again simulation results previously obtained for the PGZ1-experiment^9^.

### The module scale

#### Simulations with P_c,e_ = 0 and benchmarking

**Figure 10:**
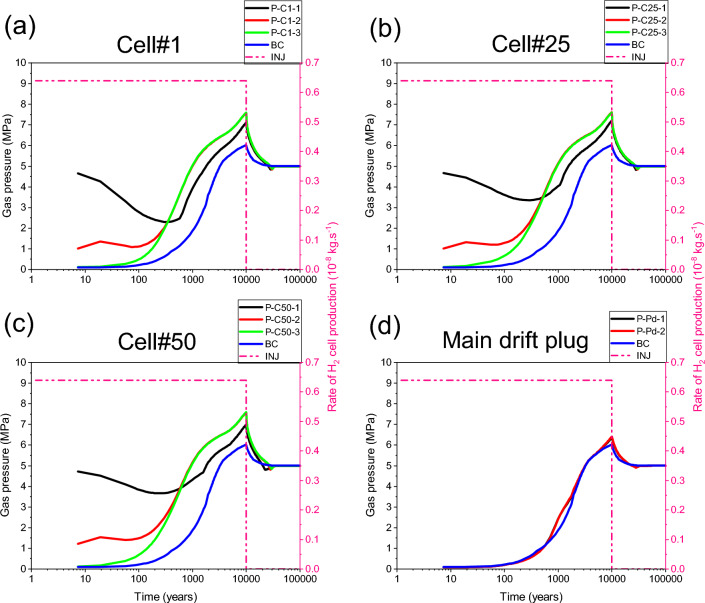
Evolutions in time of gas pressure simulated by a parametrization P_c,e_ = 0. (**a**, **b**, **c**) Points P–C#-1,2,3 (#: n° of the cell). (**d**) Points P-Pd-1,2 in the main drift plug (Fig. 2). BC: boundary condition; INJ: injected mass of hydrogen per unit time, generated by the corrosion of canisters of the disposal waste cell during 10 000 y.

**Figure 11:**
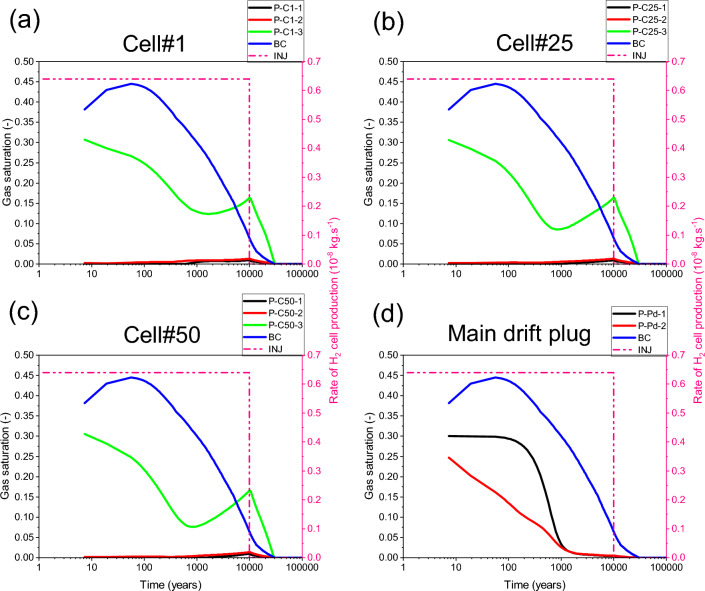
Evolutions in time of gas saturation simulated by a parametrization P_c,e_ = 0. (**a**, **b**, **c**) Points P–C#-1,2,3 (#: n° of the cell). (**d**) Points P-Pd-1,2 in the main drift plug (Fig. 2). BC: boundary condition; INJ: injected mass of hydrogen per unit time, generated by the corrosion of canisters of the disposal waste cell during 10 000 y.

Our numerical solution converges for the whole simulation period of 100 000 y. Figure 10a,b,c shows time-varying gas pressure at the three points P-C1-3, P-C25-3 and P-C50-3 located in the access drift, along axes of cells #1, #25, #50 (Fig. 2), respectively, which were initially unsaturated (S_g,ini_ = 0,3). The gas pressure increases significantly in the access drift and achieves its maximum (~ 7.57 MPa) at the downtime of gas production by cells (10 000 y). The gas saturation evolves in the same way at each of these points (Fig. 11a,b,c), showing a partial water re-saturation of the drift during ~ 1000 y (S_g_ = 0,1), then its water desaturation following the gas pressure build-up to achieve S_g_ = 0,15 at 10 000 y, and finally its total water re-saturation at about ~ 30 000 y (S_g_ = 0) which corresponds to the transit time to the hydrostatic equilibrium in the drifts network (blue curves BC in Figs. 10 and 11 : Time-dependent Dirichlet boundary condition at the main drift exit).

For the points P-C1-2, P-C25-2, and P-C50-2 situated in the homogenized EDZ-interface, which were initially unsaturated, gas pressure curves (Fig. 10a,b,c) follow the same trend in time as in points located in the backfilled access drift (P-C#-3, # = 1, 25, 50), except at early times, during the first 250 y. However, due to the retention properties of the homogenized layer EDZ-interface (like COx), gas saturation at these points elements (P-C#-2, # = 1, 25, 50) is much smaller than those in the backfilled acces drift, and on the other it is like that at points P-C1-1, P-C25-1, and P-C50-1 located in the COx (5 m behind the cells, Fig. 2), initially fully water saturated (S_g_ = 0). Points in the COx begin to desaturate slightly after ~ 1000 y, due to gas advance in the COx (Fig. 11a,b,c) during the period of significant H_2_ production by cells. This time evolution is the result of the significant gas pressure evolution at these points (Fig. 10a,b,c), where their maximum values achieve ~ 7.0 to 7.2 MPa (according to their positions from the access drift exit).

The gas pressure evolves in time in the same way at points P-Pd-1 et P-Pd-2 (overlap of grey and red curves in Fig. 10d) initially unsaturated (S_g,ini_ = 0.3), located in the bentonite-plug of the main drift (Fig. 2). This time-evolution, due to H_2_ gas front advance in the main drift from both sides of the bentonite-plug, does not highly differ from that imposed at the exit of the main drift (blue curve: BC). The gas originating from the exit BC slightly increases the maximum gas pressure simulated at 10 000 y (~ 6.3 MPa). However, the gas saturation degree evolves differently at these two points (Fig. 11d). The point P-Pd-2 saturates prematurely, prior to point P-Pd-1, due to its proximity to the saturated COx. Ultimately, these evolutions show that the bentonite-plug in the main drift has undergone significant and rapid water re-saturation after ~ 1000 y (S_g_ = 0.03), then a low and very slow water re-saturation after the arrival of gas front, reaching then the minimum value S_g_ = 1 × 10^–5^ at time t ~ 28 000 y. The full water re-saturation (S_g_ = 0) of the plug is achieved prior to the transit time to hydrostatic equilibrium (30 000 y), which explains the key role that plays this material in the reduction of gas flux that could reach the exit of the main drift.

**Figure 12:**
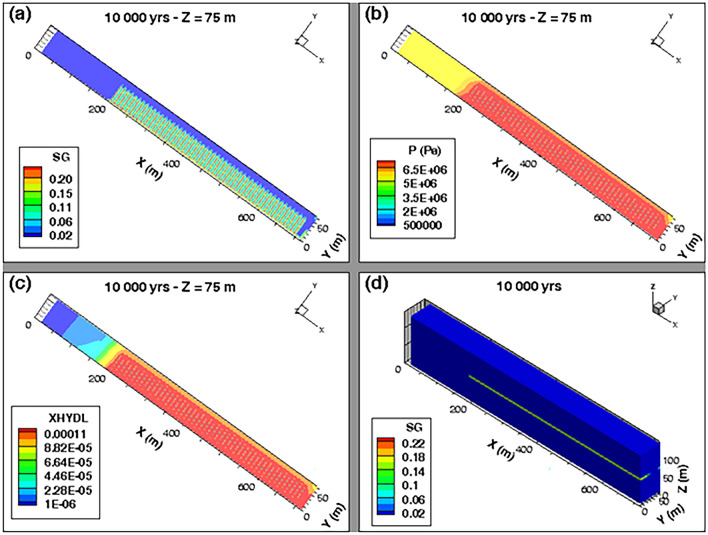
Profiles of the state variables simulated at time t = 10 000 y in the module inside the COx with a parametrization P_c,e_ = 0. 2D view in plane XY at slice plane Z = 75 m of: (**a**) Gas saturation degree, (**b**) Pressure and (**c**) Mass fraction of dissolved H_2_ in water. (**d**) 3D view of the gas saturation degree in the whole domain.

The 2D profile of gas saturation degree (Fig. 12a) at slice plane Z = 75 m (vertical mid-height of the COx layer), simulated at time t = 10 000 y, shows a significant desaturation of backfilled drifts and COx around cells. A zoom on some cells near the main drift (see Supplementary Fig. S2b online) shows that an unsaturated zone with high gas saturation (red zone with S_g_ ~ 0,2) is developed in the EDZs of backfilled drifts and cells. Notice, however, the full water re-saturation of the main drift plug with respect to the other backfilled parts of the drifts network (see also 3D profile of S_g_ in Fig. 12d, and curves S_g_(t) in points P-Pd-1 and P-Pd-2 in Fig. 11d). The 2D profile of S_g_ at slice plane y = 3 m (exit of cells plugs onto the access drift), shown in the Supplementary Fig. S2c online, confirms this result. Within the access drift at slice plane y = 2.5 m (see Supplementary Fig. S2a online), S_g_ is much higher.

The 2D profile of S_g_ along the cell axis at slice plane x = 690.5 m, shown in the Supplementary Fig. S2d online, explains well the low desaturation of the COx zone behind the cells, as also shown in Fig. 11a (point P-C1-1).

The 2D profile of pressure (Fig. 12b) at slice plane Z = 75 m, simulated at time t = 10 000 y, shows development of a red zone surrounding all the cells and which is at a pressure of ~ 7 MPa. Beyond that zone area, an advance of the front of water in both directions OX and OY is simulated, but without any substantial desaturation of the COx. This advance along the X-axis of about 100 m is due to the important solubility of hydrogen in the COx, confirmed as well by the 2D profile of the mass fraction of H_2_ dissolved in water, simulated at slice plane Z = 75 m at 10 000 y (Fig. 12c).

The maximum gas pressure obtained by our model at point P-C25-3 at t ~ 10 000 y (7.57 MPa, Fig. 10b) doesn’t differ from that obtained by^27^ (see Fig. 11 of^27^) who used TOUGH2-MP/EOS5 with LaGriT mesh^31^ and homogenization of interfaces with fractions of their adjacent materials as well. Moreover, our model simulates a maximum gas pressure of about 6.3 MPa at point P-Pd-2 (Fig. 10d) which is slightly higher than the one simulated by these authors (5.8 MPa, see Fig. 12 of^27^). However, some discrepancies were simulated during the gas pressure evolution at the beginning of ~ 3000 y before maximum gas pressure achievement. In fact, the gas pressure increase from this time is much slower with our model. These discrepancies can be explained by the differences between meshes, between the models of the diffusion coefficient of H_2_ in a two-phase porous medium, and between methods of the homogenization of interfaces with adjacent materials, used by each team modelers.

Also notice that^32^ simulated a maximum gas pressure of ~ 7.8 MPa at point P-C25-3 and of ~ 6.8 MPa at points P-PU-1 and P-PU-2 (upstream points rather than downstream points P-Pd-1 and P-Pd-2), which are approximately the same as those simulated by our model. Recall here that his^32^ simulations were carried out using a rectilinear and structured mesh by considering explicitly the centimetric interfaces.

#### Simulations with P_c,e_ ≠ 0

**Figure 13:**
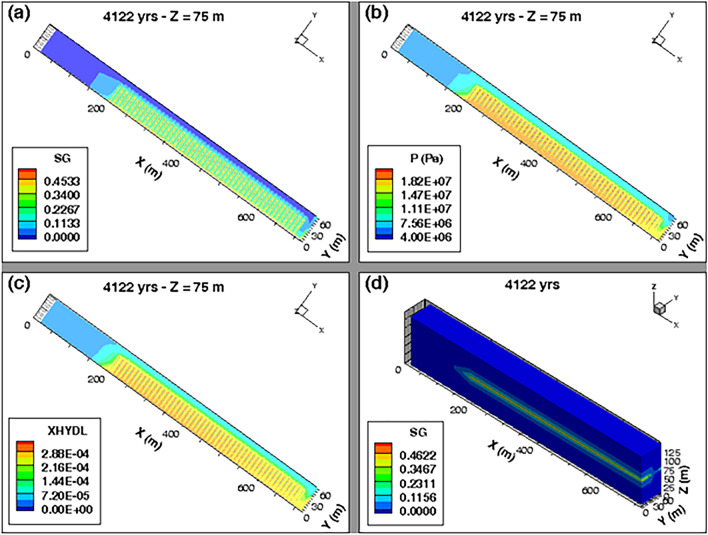
Profiles of state variables simulated at time t = 4122 y in the module inside the COx with a parametrization P_c,e_ ≠ 0. 2D view in plane XY at slice plane Z = 75 m of: (**a**) Gas saturation degree, (**b**) Pressure, and (**c**) Mass fraction of dissolved H_2_ in water. (**d**) 3D view of the gas saturation degree in the whole domain.

Numerical simulations with a parametrization P_c,e_ ≠ 0 did not achieve 100 000 y because of a numerical divergence of the solution at time t ~ 4122 y. When this numerical divergence occurred, the gas pressure build-up in the backfilled access drift (points P-C1-3, P-C25-3, P-C50-3) was very high (more than 18 MPa). The gas saturation degree in the backfill achieved ~ 0,42. These results are confirmed by the profile of gas saturation degree S_g_ (Fig. 13a,d), and profile of liquid or gas pressure P_l_ or P_g_ at slice plane Z = 75 m (Fig. 13b), simulated at time t = 4122 y. The S_g_-profile at slice plane Z = 75 m (Fig. 13a) also show an unsaturated zone of high gas saturation degree, which envelops all the cells, and which induces a significant advance of an unsaturated front of water in the COx zone over a distance of ~ 50 m along X-axis for X between 150 and 200 m (gas ‘piston effect’,^15^). This desaturation of the COx is essentially due to the high pressure of H_2_ which propagates from the backfilled drifts to the COx (yellow zone in Fig. 13b) leading to an important advance of the dissolved H_2_ front in water (Fig. 13c). A zoom on pressure profiles near cells (see Supplementary Fig. S3b,d online) and in the access drift (see Supplementary Fig. S3a,c online) confirm these results. These results can be explained by:

- The mesh, initially developed for parametrization P_c,e_ = 0 (Fig. 4), is perhaps not adequate for simulating high pore gas velocities in the access drift, which can be higher than 10^–5^ m/s in some zones of the domain. Therefore, new meshes must be developed and tested to avoid the high gas pressure gradients in these regions for a parametrization P_c,e_ ≠ 0. This new mesh should comply to a maximal Peclet number for all mesh elements with these high pore gas velocities to reduce the numerical dispersion. Moreover, these high gas velocities violate the validity domain of Darcy’s law which is initially developed for laminar flows with slow gas velocities.
- The significant H_2_ pressure calculated in the backfilled drifts (~ 18 MPa) induces a significant desaturation of the COx with an increase of gas pressure within it (see Supplementary Fig. S3 online). This H_2_ pressure far exceeds the lithostatic (fracturing) pressure 11.3 ± 0.3 MPa of the COx indicated by Andra^7^. This pressure build-up can trigger a micro-fissuring of the COx due to pores dilation, as well as an opening of preferential flow paths which are not accounted by the current model. These micro-fissures and/or fractures can present an escape route for gas in the COx in high quantities, but at the expense of the COx structure changing.

The important accumulation of H_2_ in the drifts is due to its preferential transport in the homogenized interfaces, backfill, and EDZ, having the smallest P_c,e_-values. The COx, having the highest P_c,e_-value, plays the role of a capillary barrier which does not allow at the beginning creation of two-phase flow pathways in the COx and blocks temporarily the gas phase in the drift until its pressure builds up outer in the COx and exceeds the fracturing pressure of the COx (11.3 ± 0.3 MPa). This mechanism is likely accentuated by the presence of the bentonite-plug in the main drift.

It is worth mentioning, that attempts exist to control the unwanted pressure buildup in form of an Engineered Gas Transport System (EGTS) by modifying essentially the seals and the backfill materials (e.g. bentonite-plug in the main drift, Fig. 2) as proposed by Nagra^33^.

**Figure 14:**
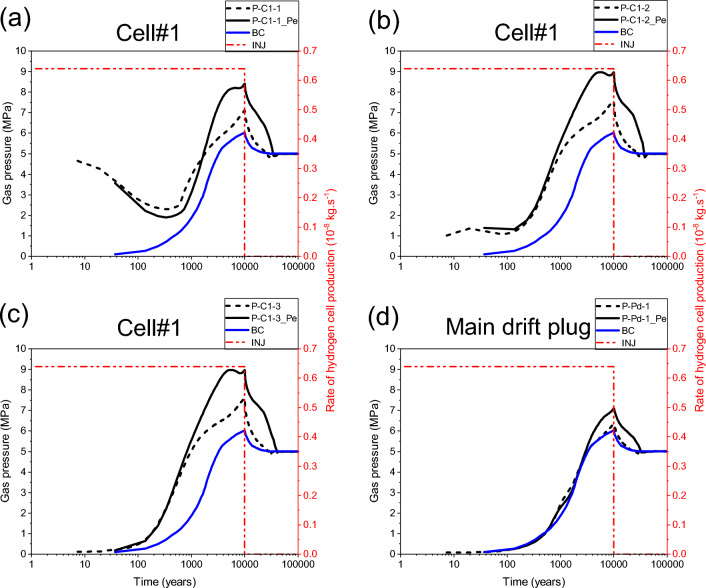
Evolutions in time of gas pressure simulated by parametrizations P_c,e_ = 0 and P_c,e_ ≠ 0. (**a**, **b**, **c**) Points P–C#-1,2,3 (#: n° of the cell). (**d**) Points P-Pd-1,2 in the main drift plug (Fig. 2). BC: boundary condition; INJ: injected mass of hydrogen per unit time, generated by the corrosion of canisters of the disposal waste cell during 10 000 y.

**Figure 15:**
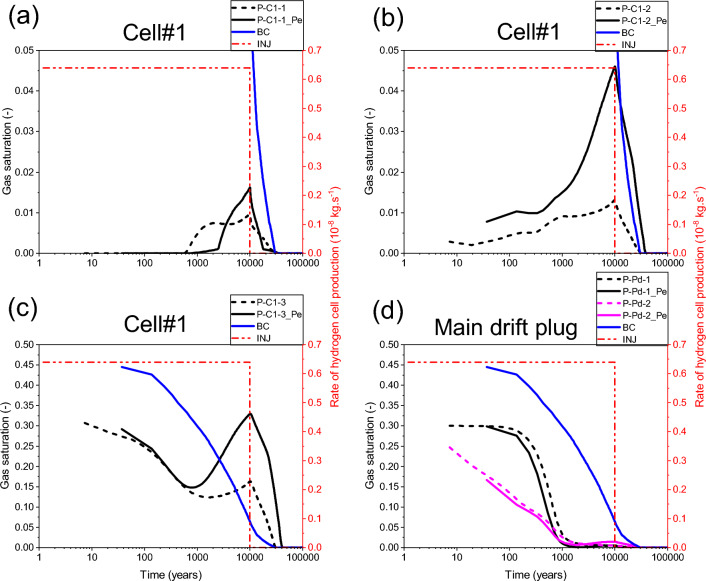
Evolutions in time of gas saturation simulated by parametrizations P_c,e_ = 0 and P_c,e_ ≠ 0. (**a**, **b**, **c**) Points P–C#-1,2,3 (#: n° of the cell). (**d**) Points P-Pd-1,2 in the main drift plug (Fig. 2). BC: boundary condition; INJ: injected mass of hydrogen per unit time, generated by the corrosion of canisters of the disposal waste cell during 10 000 y.

As an alternative to SGM-solution which is difficult to converge for no more than 4122 y at the module scale for parametrization P_c,e_ ≠ 0, simulations have been performed with an approximative solution using a regularization technique for P_c_(S_l_) (Eq. (1)) linearization near full liquid saturation. Although this approximation can underestimate the real maximum gas pressure within the drift, it can help to explain how P_c,e_ can impact, through the VGMPE model, the different transport pathways of H_2_ by diffusion and by convection in the module during 100 000 y. By using this approximation, the gas pressure simulated by parametrization P_c,e_ ≠ 0 increases in all points elements by comparison to that simulated with parametrization P_c,e_ = 0. Figure 14 shows this comparison for the example of points P-C1-1, P-C1-2, P-C1-3 near Cell#1 and for points P-Pd-1 and P-Pd-2 in the main drift plug. The maximum gas pressure (peak achieved at ~ 10 000 y) at the three points elements P-C1-1, P-C1-2, P-C1-3 increases by about 1.5 MPa (Fig. 14a,b,c), while that at points elements P-Pd-1 and P-Pd-2 increases by about 1 MPa (Fig. 14d). This increase in gas pressure is also accompanied by a substantial increase of water drainage and gas saturation in the EDZ (point P-C1-2_Pe, Fig. 15b), and access drift (point P-C1-3_Pe, Fig. 15c).

For the point P-C1-1 located in the clay host rock (COx), Fig. 15a, there is a premature desaturation of the COx during time when parametrization P_c,e_ = 0 is considered. However, for parametrization P_c,e_ ≠ 0, although desaturation of the COx is delayed, its magnitude is higher than that is simulated by the parametrization P_c,e_ = 0. This result is like that obtained by^9^ in their study of nitrogen gas migration in the COx by using both parametrizations. However, this result differs from that is simulated in the clay host rock at the cell scale (Fig. 9) for parametrization P_c,e_ ≠ 0. This is explained by the high gas pressure that is involved at module scale in the EDZ, and access and main drifts due to higher P_c,e_-values of the COx and bentonite-plug that leaded to the accumulation of gas and its confinement in the materials with low P_c,e_ values (EDZ, backfill).

Notice also in Fig. 15 a delay of about 10 000 y before reaching hydrostatic equilibrium (re-saturation) at all points in the EDZ and backfilled drifts after time 10 000 y when parametrization P_c,e_ ≠ 0 is considered.

**Figure 16:**
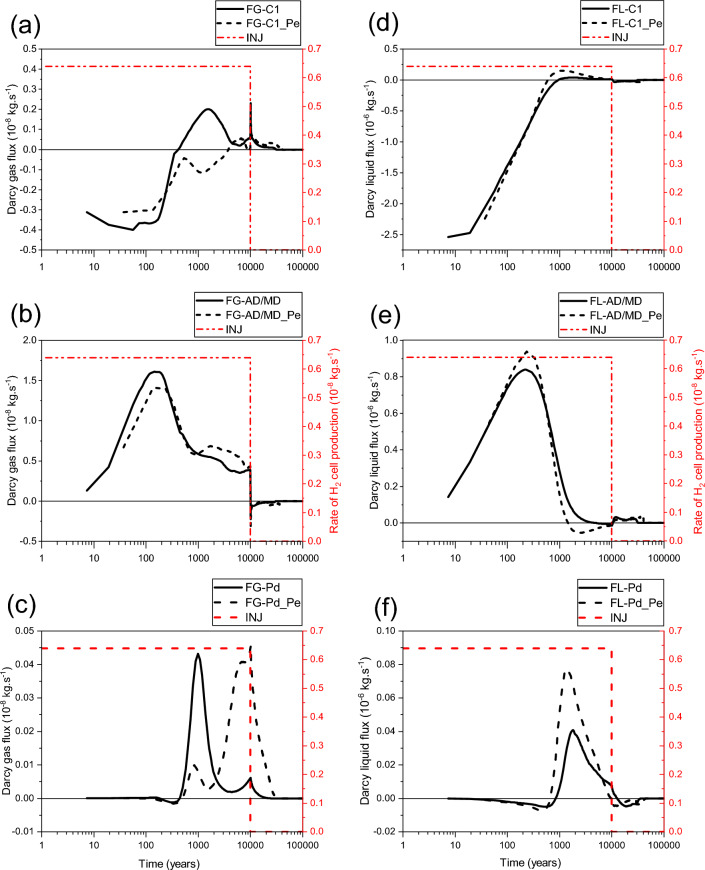
Evolutions in time of Darcy flux simulated at the three cross sections F-C1, F-AD/MD and F-Pd (Fig. 2) by using both parametrizations P_c,e_ = 0 and P_c,e_ ≠ 0. (**a**, **b**) F-C1; (**c**, **d**) F-AD/MD; (**e**, **f**) F-Pd. (**a**, **b**, **c**) Gas phase; (**d**, **e**, **f**) Liquid phase. INJ: injected mass of hydrogen per unit time, generated by the corrosion of canisters of the disposal waste cell during 10 000 y.

There is a fast re-saturation of the main drift bentonite plug (fast decrease of gas saturation, Fig. 15d) due to the increase of water flux in the plug (Fig. 16f) in the case of parametrization P_c,e_ ≠ 0. Moreover, the fact that bentonite-plug has an important P_c,e_-value greater than that of the backfill of the drift, explains this increase.

The analysis of the results for points elements P-C25-# (# = 1,2,3) and P-C50-# (# = 1,2,3) (not shown here, just for avoiding many figures in the text) is like to that for points elements P-C1-# (# = 1,2,3).

**Figure 17:**
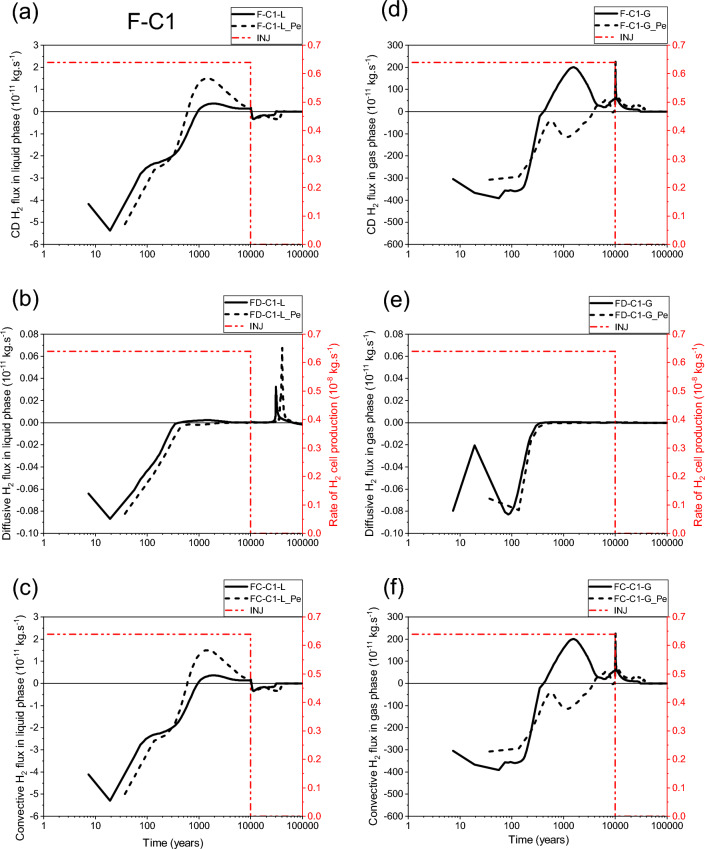
Evolutions in time of H_2_ flux simulated at cross section F-C1 (Fig. 2) by using both parametrizations P_c,e_ = 0 and P_c,e_ ≠ 0. (**a**, **d**) Convection–Diffusion (CD); (**b**, **e**) Diffusion (D); (**c**, **f**) Convection (C). (**a**, **b**, **c**) Liquid phase; (**d**, **e**, **f**) Gas phase. INJ: injected mass of hydrogen per unit time, generated by the corrosion of canisters of the disposal waste cell during 10 000 y.

As shown by curves in Fig. 17a,b,c, there is an important convective transport of dissolved H_2_ in liquid water from the homogenized EDZ-interface material to the bentonite plug in the disposal waste cell (FC-C1-L and FC-C1-L_Pe, Fig. 17c) during the first ~ 600 y and first 1000 y, for both parametrizations, P_c,e_ ≠ 0 and P_c,e_ = 0, respectively. However, beyond these short periods, a return convective transport of dissolved H_2_ towards the homogenized EDZ-interface material is simulated until the gas production ceases at 10 000 y and the cell plug begins to re-saturate. This return flow is more important in the case of parametrization P_c,e_ ≠ 0. Beyond 10 000 y, this transport is again directed towards the cell plug during a period of about 20 000 y for parametrization P_c,e_ = 0, and 10 000 y later for parametrization P_c,e_ ≠ 0 before vanishing at hydrostatic equilibrium.

For both parametrizations (P_c,e_ ≠ 0 and P_c,e_ = 0), Fig. 17d,e,f, there is a convection-dominated transport in the gas phase from the homogenized EDZ-interface material towards the bentonite plug in the disposal waste cell (FC-C1-G and FC-C1-G_Pe, Fig. 17f). However, a return transport is simulated earlier for parametrization P_c,e_ = 0 (FC-C1-G at ~ 400 y) than that is simulated for parametrization P_c,e_ ≠ 0 (FC-C1-G_Pe at ~ 3830 y). Both fluxes FC-C1-G and FC-C1-G_Pe become smaller after ~ 4500 y. The flux FC-C1-G vanishes earlier at ~ 30 000 y when a full water saturation is achieved at the main drift exit, whereas it continues for an additional duration of ~ 10 000 y (till time ~ 40 000 y) for the flux FC-C1-G_Pe, before reaching hydrostatic equilibrium, Fig. 15c.

**Figure 18:**
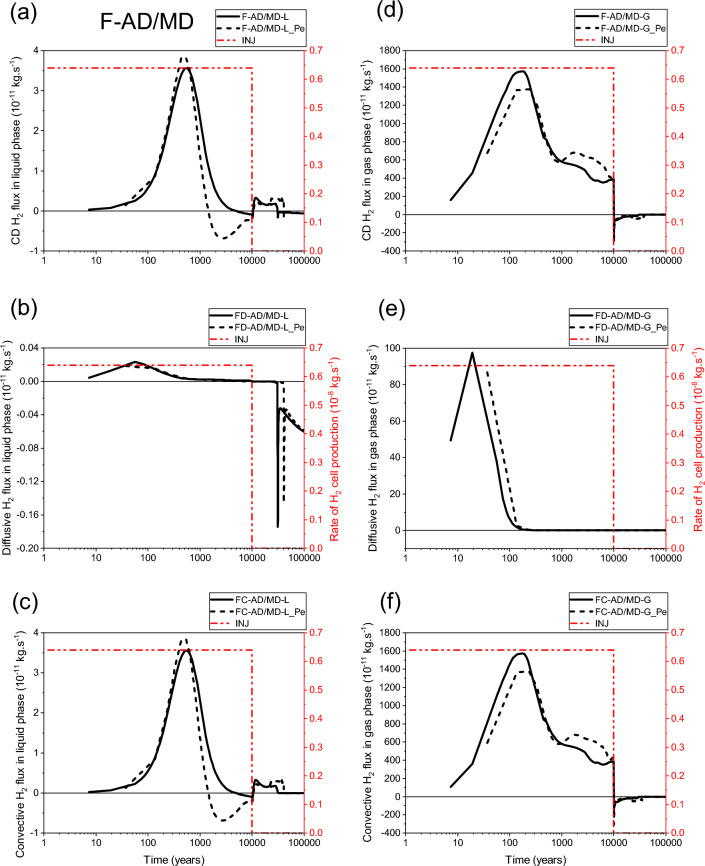
Evolutions in time of H_2_ flux simulated at cross section F-AD/MD (Fig. 2) by using both parametrizations P_c,e_ = 0 and P_c,e_ ≠ 0. (**a**, **d**) Convection–Diffusion (CD); (**b**, **e**) Diffusion (D); (**c**, **f**) Convection (C). (**a**, **b**, **c**) Liquid phase; (**d**, **e**, **f**) Gas phase. INJ: injected mass of hydrogen per unit time, generated by the corrosion of canisters of the disposal waste cell during 10 000 y.

For both parametrizations (P_c,e_ = 0 and P_c,e_ ≠ 0), the simulated transport of dissolved H_2_ in liquid water by diffusion towards the AD-exit (fluxes FD-AD/MD-L and FD-AD/MD-L_Pe, Fig. 18b) is negligible compared to that simulated by convection (fluxes FC-AD/MD-L and FC-AD/MD-L_Pe, Fig. 18c). There is a parallel increase of both fluxes, FC-AD/MD-L and FC-AD/MD-L_Pe, towards the main drift during the first ~ 700 y (with a very small difference, even between peaks). Subsequently, these fluxes decreased differently until a return transport towards the access drift is produced. This return is more significant and much larger in time for FC-AD/MD-L_Pe, which started earlier at ~ 1500 y than for FC-AD/MD-L at ~ 5000 y, until the gas production stops at 10 000 y. Beyond these times, both fluxes FC-AD/MD-L and FC-AD/MD-L_Pe become small, positive (flux towards the main drift) and practically constant in time (~ 0.2 to 0.3 × 10^–11^ kg·s^−1^) before vanishing at ~ 30650 and ~ 40770 y, respectively (delay of ~ 10 120 y). This delay is like that simulated by FC-C1-L_Pe (Fig. 17c), showing the importance of the capillary gas entry pressure in delaying the transport of dissolved H_2_ in the liquid phase near the disposal waste cell and drift network of the domain, even when the exit boundary becomes saturated at ~ 30 000 y.

Although the H_2_ diffusive flux in the gas phase towards the AD-exit simulated by parametrization P_c,e_ ≠ 0 (FD-AD/MD-G_Pe) overestimates that simulated by parametrization P_c,e_ = 0 (FD-AD/MD-G), the difference between them is short in time (~ 170 y), Fig. 18e. Moreover, their contribution to the CD-flux (F-AD/MD-G, Fig. 18d) beyond 170 y is very negligible compared to convective fluxes in gas phase (Fig. 18f).

For both parametrizations, P_c,e_ ≠ 0 and P_c,e_ = 0, there is an important convective transport of H_2_ in the gas phase towards the AD-exit during the whole period of gas generation (Fig. 18f). Notice that FC-AD/MD-G_Pe underestimates FC-AD/MD-G during a short period of ~ 230 y. However, beyond ~ 1000 y, FC-AD/MD-G_Pe overestimates FC-AD/MD-G during a long period which is ~ 9000 y, until the gas production stops at 10 000 y. After 10 000 y, both simulated convective fluxes behave similarly in time with a return transport of H_2_ towards the main drift until vanishing (with a delay of ~ 10 000 y for FC-AD/MD-G_Pe vanishing at t ~ 40 000 y).

**Figure 19:**
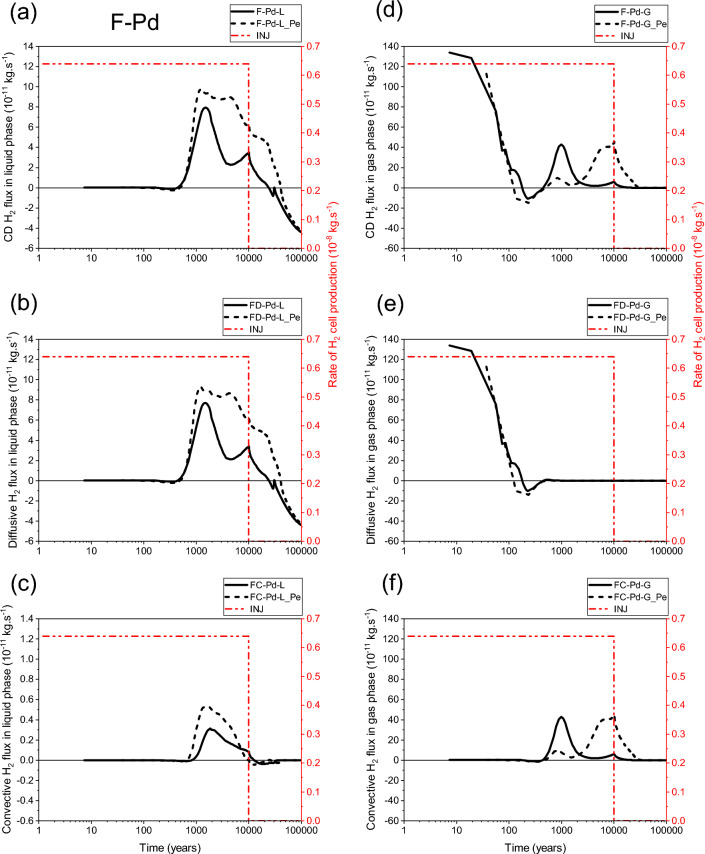
Evolutions in time of H_2_ flux simulated at cross section F-Pd (Fig. 2) by using both parametrizations P_c,e_ = 0 and P_c,e_ ≠ 0. (**a**, **d**) Convection–Diffusion (CD); (**b**, **e**) Diffusion (D); (**c**, **f**) Convection (C). (**a**, **b**, **c**) Liquid phase; (**d**, **e**, **f**) Gas phase. INJ: injected mass of hydrogen per unit time, generated by the corrosion of canisters of the disposal waste cell during 10 000 y.

Unlike FD-AD/MD-L and FD-C1-L, there is a diffusion-dominated transport of dissolved H_2_ in liquid phase in the main drift bentonite plug during the whole period of the simulation (100 000 y) for both parametrizations (P_c,e_ = 0 and P_c,e_ ≠ 0), Fig. 19a,b,c. Notice, however, that the diffusive flux FD-Pd-L_Pe is usually higher than FD-Pd-L. This can be explained by the accumulation of H_2_ in the backfill of the main drift due to its small P_c,e_-value compared to that of the bentonite plug. This result is in accordance with that obtained for the disposal waste cell (Figs. 6 and 9), for the diffusive transport of dissolved H_2_ in the COx from the EDZ. This diffusive transport is first directed towards the main drift exit BC during the periods ~ 25 000 y and ~ 40 000 y for parametrizations P_c,e_ = 0 and P_c,e_ ≠ 0, respectively, before a return diffusive transport towards the access drift begins when the exit boundary begins to be fully water saturated.

Figure 19c shows that the convective transport of dissolved H_2_ in liquid phase simulated by both parametrizations, although negligible compared to that of the diffusion (Fig. 19b), is directed towards the exit BC during almost all the period of gas generation (10 000 y). Notice also that FC-Pd-L_Pe is higher than FC-Pd-L during this period, but both fluxes decrease by increasing water saturation at the exit boundary until reaching small return flow rates from the plug towards the access drift before vanishing entirely at ~ 32 000 y and ~ 42 000 y for parametrizations P_c,e_ = 0 and P_c,e_ ≠ 0, respectively (delay of about 10 000 y).

During the first ~ 500 y, H_2_ is mainly transported in the drift plug by diffusion in the gas phase towards the main drift exit BC (Fig. 19e). There is a very small difference between fluxes calculated by both parametrizations. Notice a return diffusive transport towards the access drift simulated after a short period (~ 100 and ~ 200 y for P_c,e_ ≠ 0 and P_c,e_ = 0, respectively) before entirely vanishing. Beyond ~ 500 y there is a convection-dominated transport towards the exit BC until hydrostatic equilibrium is reached (full liquid saturation). Although this flux is smaller than that due to diffusion-only, it is larger in time. The most important result is that the first significant peak of H_2_ simulated by parametrization P_c,e_ = 0 (FC-Pd-G ~ 43 × 10^–11^ kg·s^−1^) appears earlier, but it is short in time (period of ~ 1810 y between times ~ 440 and ~ 2250 y; after which it becomes very small), whereas the second significant peak of H_2_ simulated by parametrization P_c,e_ ≠ 0 (FC-Pd-G_Pe ~ 41 × 10^–11^ kg·s^−1^) appears later, but it is large in time (period of ~ 27,750 y between times 2250 and 30 000 y). The latter peak vanishes until the exit BC becomes saturated. This shows that mass of hydrogen reaching the drift plug, and thus the exit BC, through gas phase, by using parametrization P_c,e_ ≠ 0 is more significant than that is simulated by using parametrization P_c,e_ = 0. This delay, as well as significant H_2_ mass reaching the plug simulated by parametrization P_c,e_ ≠ 0 are the consequences of higher P_c,e_ values of clay host rock (COx) and drift plug bentonite (2 and 1.33 MPa, respectively, Table 1) which do not ease the escape of the cumulated gas in the backfilled drift network, and therefore lead to the increase of gas pressure and gas flow within this network. Gas entry in the drift plug is delayed until the difference between gas pressure in the drift backfill and liquid pressure in the drift plug becomes higher than the P_c,e_-value of the drift bentonite plug.

The significant convective gas transport towards the main drift plug and the exit BC for parametrization P_c,e_ ≠ 0, explains the importance of gas piston effect on water flow shown in Fig. 16f for this parametrization.

## Conclusion

This study is the first one to show the impact of a non-zero gas-entry pressure on the results of gas migration modeling in the various components of a DGR (cell and module). Illustration of this impact through FORGE benchmark models for both HLW cell and module shows that gas accumulates mainly in materials having the lowest P_c,e_ values (interfaces, EDZ and backfill), which can substantially increase the gas pressure within the backfilled drift network and its neighborhood (host rock). In the case of FORGE benchmarks, the gas pressure can reach very high values, thus taking the model out of its range of validity and requiring additional complex hydromechanical processes to be considered, such as pathway dilations, opening of interfaces or cracking/fracturing of the host rock.

Enhancement of the numerical modelling of gas migration within the different components of a DGR may also require consideration of processes other than the gas-entry pressure, such as hysteresis, thermal, chemical, and hydro-gas-mechanical processes. These processes might influence porosity and pore size distribution, which would change parameterization of capillary-pressure—saturation—permeability—diffusivity relations.

Finally, all these enhancements could help in future studies to evaluate carefully advective and diffusive transport of radionuclides within the backfilled drifts.

### Supplementary Information

Supplementary Figures.

## Data Availability

All data that support the findings of this study are available from the corresponding author on request.
