# Supplementary material for: Gas-entry pressure impact on the evaluation of hydrogen migration at different scales of a deep geological disposal of radioactive waste
Source: Sci Rep. 2024 Mar 14;14:6221. doi: 10.1038/s41598-024-56454-y (PMC10940707; doi:10.1038/s41598-024-56454-y)

Gas-entry pressure impact on the evaluation of hydrogen migration at different scales of a deep geological disposal of radioactive waste

Zakaria Saâdi

Figure S1. Position of the studied module and the domain boundary within the repository of HLW.

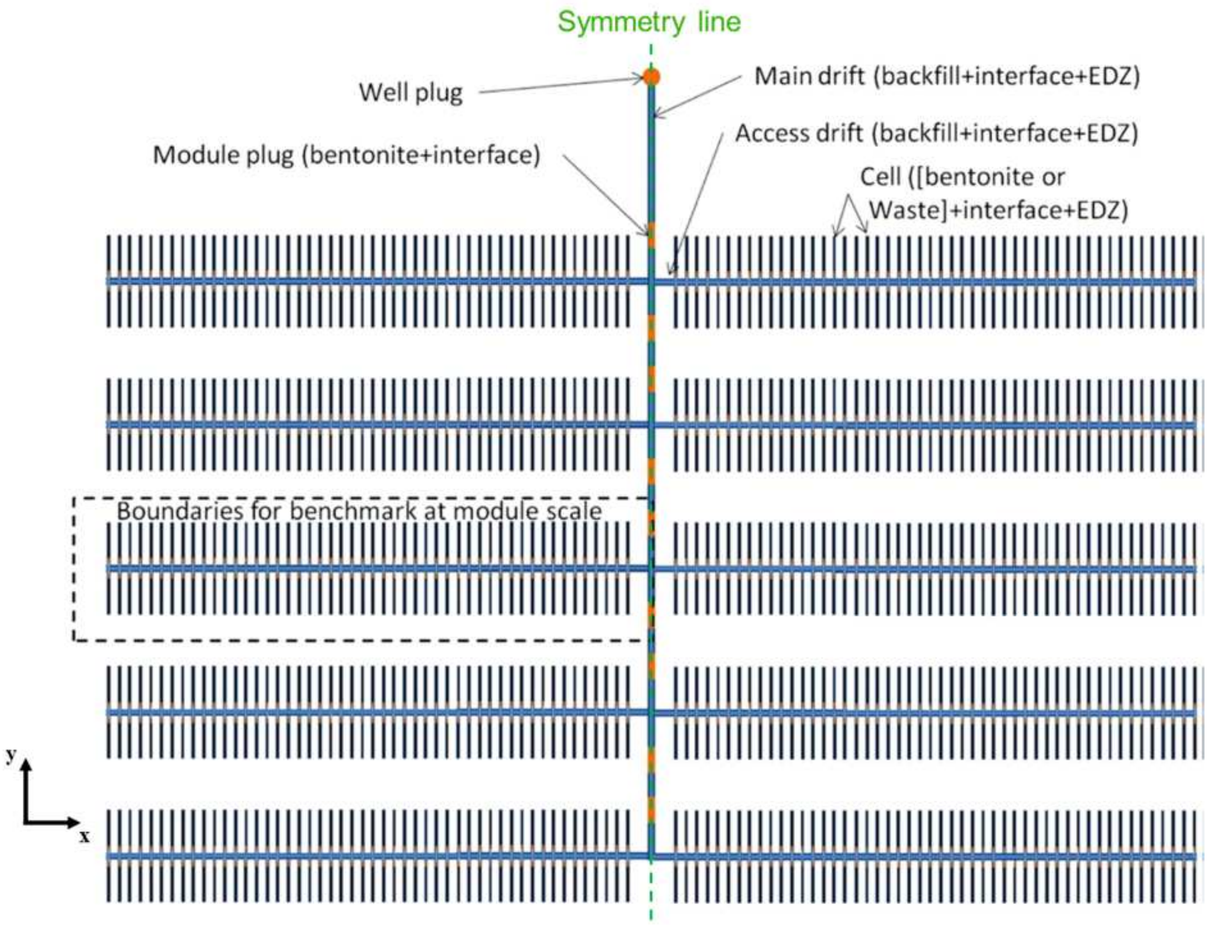

Figure S2. The 2D slices of gas saturation degree simulated by SGM scheme at time t = 10 000 y with a parametrization $P_{c,e} = 0$: (a) access drift, (b) around cells near the main drift, (c) interface cells-access drift, and (d) along axis of cell#1, the nearest cell to the main drift.

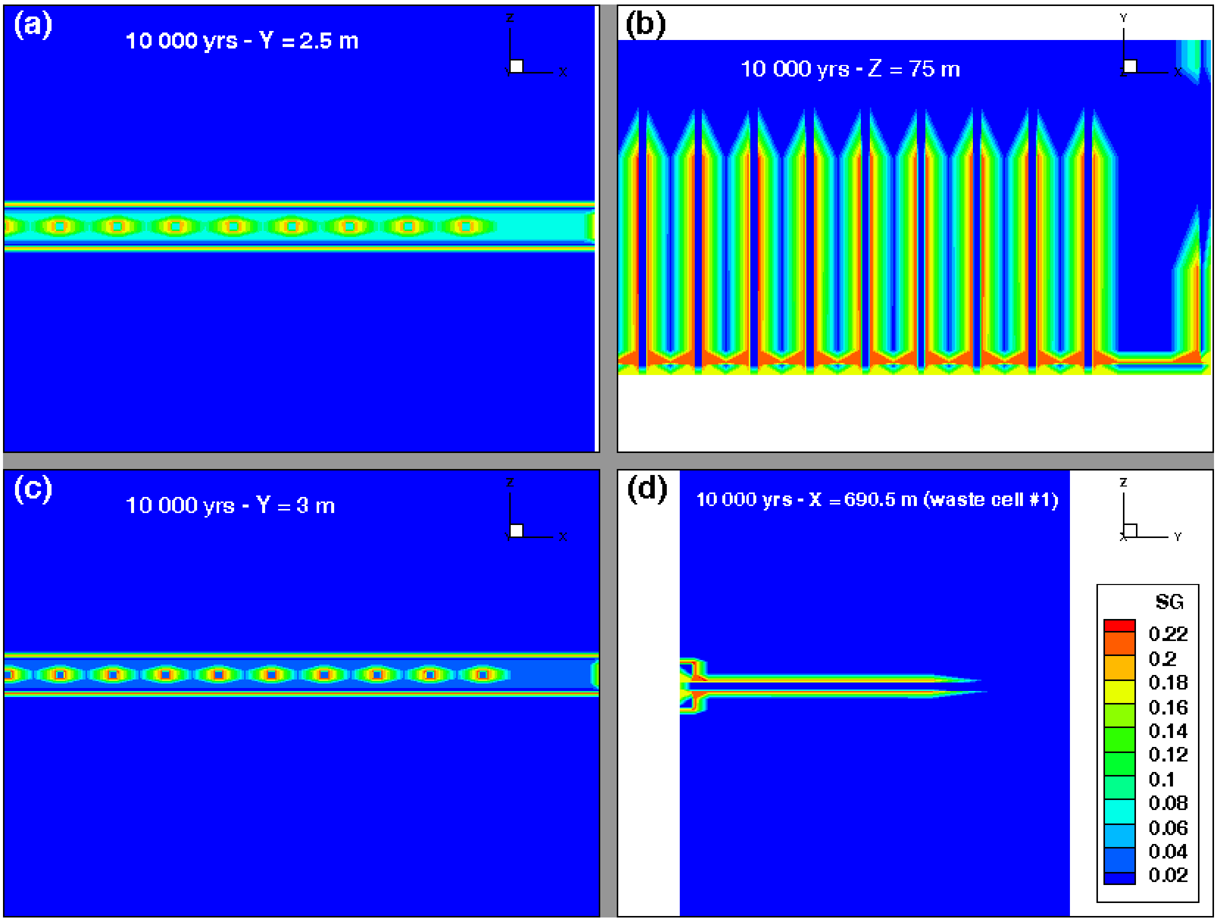

Figure S3. The 2D slices of pressure simulated by SGM scheme at time t = 4122 y with a parametrization $P_{c,e} \neq 0$: (a) access drift, (b) around cells near the main drift, and (c) interface cells-access drift, and (d) along axis of cell#1, the nearest cell to the main drift.

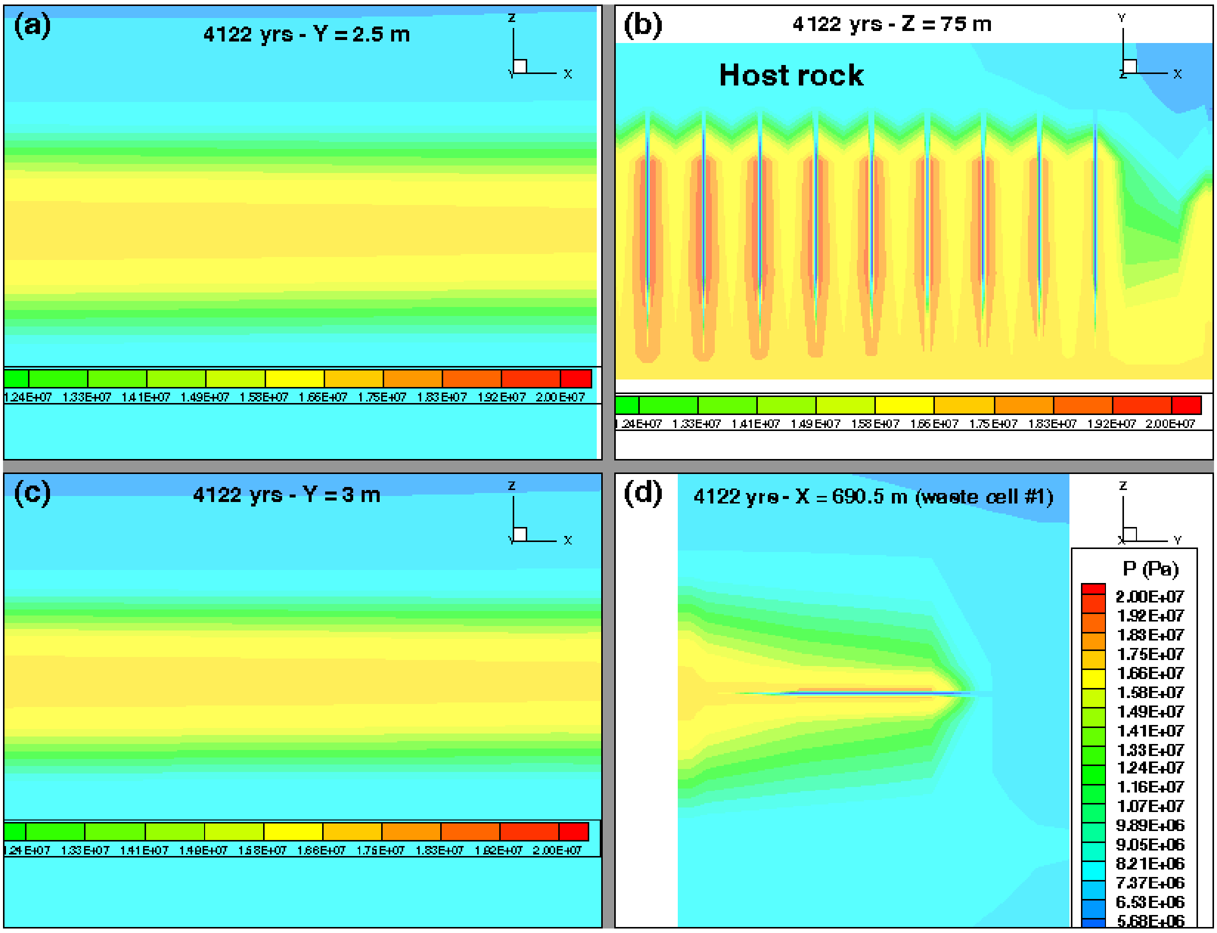

Supplement: Supplementary file 1 — Supplementary Figures. [file 41598_2024_56454_MOESM1_ESM.pdf]
